# Robotic Plasma System for Rapid Activation of Mass-Produced Electrochemical Sensors

**DOI:** 10.1021/acs.analchem.5c04556

**Published:** 2025-12-01

**Authors:** Marina Di-Oliveira, Mariana C. Marra, Raquel G. Rocha, Teodoro R. Terra, Rodrigo A. A. Muñoz, Eduardo M. Richter

**Affiliations:** Institute of Chemistry, 28119 Federal University of Uberlândia, 38408-100 Uberlândia, Brazil

## Abstract

Polishing, cleaning, and activation of solid electrode surfaces are common in electrochemistry; however, they are often labor-intensive, operator-dependent, and poorly reproducible. In the context of large-scale production of disposable solid-state sensors, such as screen-printed electrodes (SPEs), traditional surface conditioning procedures have proven to be impractical and incompatible with automated workflows. Here, we report the development of a low-cost robotic system that integrates a Tesla coil-based air-plasma generator with a Python-controlled *XY* motion platform for rapid (∼15 s per 0.11 cm^2^) and reproducible surface activation of SPEs. The main novelty of this study lies in the mechanization and reproducibility of the plasma activation process, achieved through the integration of a robotic platform with an air-plasma generator to enable standardized and scalable electrode conditioning. Morphological and Raman spectroscopic analyses revealed significant surface restructuring after plasma activation, leading to reduced charge-transfer resistance (from 7.10 to 0.45 kΩ), increased electron-transfer kinetics (∼4-fold increase in *k* ^0^), increased electroactive area (from 0.073 to 0.270 cm^2^), and improved interelectrode reproducibility (RSD from 9.7 to 0.8%). The treatment also restored the voltammetric performance of carbon, gold, and platinum SPEs stored for over ten years. Plasma-treated carbon SPEs enabled the voltammetric determination of picric acid in simulated explosive samples (linear range: 0.5–50.0 μmol L^–1^; LOD: 0.1 μmol L^–1^). Moreover, 3D-printed electrodes were also successfully treated by using this system. This robotic plasma system provides a low-cost, scalable, and ecofriendly strategy for standardized activation of disposable electrodes, enhancing or restoring their electrochemical performance for routine analytical and industrial applications.

## Graphical abstract

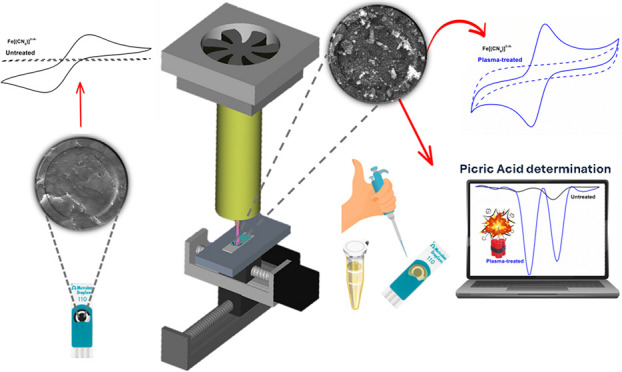

## Introduction

Laboratory automation techniques have transformed chemical analysis in recent years by replacing manual operations with robotic systems to enhance efficiency, standardize procedures, and minimize human error.^1^ Initially developed for conventional instruments such as chromatographs or spectroscopes, automated laboratory systems have evolved to address the emerging demands of miniaturization and high-throughput analysis in analytical chemistry.^2^ Nevertheless, these advancements introduce new challenges, requiring the next generation of robotic systems to offer precise control, rapid response, and high-throughput analytical capacity to ensure data reliability and scalability.^3–5^ Moreover, resource-limited regions often encounter significant barriers to accessing emerging analytical technologies.

In electroanalytical chemistry, electrode surface selection is the most important parameter, especially in the development of reliable electrochemical systems for real-time analysis and on-site monitoring. Electrode materials are expected to exhibit essential characteristics such as high selectivity, long-term operational stability, wide applicability, and chemical resistance.^6,7^ In recent years, screen-printed electrodes (SPEs) have gained increasing attention for a wide range of applications, particularly in electrochemical sensing.^8,9^ While some studies have explored the use of electroanalytical techniques for detecting various analytes in diverse sample types, the majority highlight the crucial role of electrode surface pretreatment or modification in enhancing the electrochemical activity of an electrode substrate.^10–12^

In this context, non-thermal arc-discharge plasma emerges as a powerful approach for activating various electrode surfaces. In this technique, an electron discharge generates free radicals from the atmospheric gases and cleaves the bonds between sp^2^-hybridized carbon atoms, leading to the formation of various functional groups on the surface.^13^ As a result, the overall surface energy is enhanced, and surface roughness is introduced,^14,15^ which has proven effective in improving the electrochemical performance of a variety of electrode materials, such as screen-printed,^14^ microfabricated pyrolytic carbon,^15^ graphite sheet,^16^ glassy carbon,^17^ and additively manufactured electrodes (AMEs).^18^ Despite most of them reporting an improvement in the electrochemical response of the electrode, they require bulky and expensive instrumentation.

Siqueira and collaborators carried out the electrochemical activation of AMEs using a commercial plasma pen, originally designed for dermatological applications.^19^ The plasma pen operates by exciting atmospheric gases (N_2_, O_2_, and CO_2_) by using an energy source that generates a high-voltage, high-frequency electric field. This electric field is applied between the pen’s tip (cathode) and the electrode surface (anode), enabling the activation process. During the procedure, the pen was manually guided around the electrode surface to perform the activation at an exposure rate of 631 s per cm^2^. The plasma pen demonstrated several noteworthy features, including ease of access (low-cost and widespread commercial availability), portability (battery-powered and suitable for use in various settings), and promising electrochemical performance in the activation of AMEs, enabling the detection of capsaicin in pepper sauce samples at low concentrations (LOD = 3.0 nmol L^–1^). However, this approach also presented certain challenges, including prolonged electrode treatment times and dependence on operator skills.

Tesla coils, high-voltage resonant transformers, are widely recognized as a prominent technology for plasma generation due to their ability to produce electrical discharges via air ionization. Their simplicity, visual impact, and effectiveness make them valuable components in various experimental and educational contexts. Recent studies indicate that plasma modules can serve as heating sources with the mentioned characteristics for applications in sensing, laser-based and broadband light sources.^20^ Furthermore, when combined with laser-induced breakdown spectroscopy (LIBS), plasma modules can enhance the sensitivity for the quantitative analysis of Cr and Pb.^21^

In this work, we demonstrate that a simple and low-cost Tesla coil kit can function as an arc-discharge air-plasma generator for the rapid (∼15 s per sensor) and reproducible surface activation of disposable SPEs. The proposed setup consists of a Tesla coil kit mounted on a laboratory stand and a basic *XY* motion platform that positions the SPEs, with movement controlled by Python-based software, enabling a fully robotic and operator-independent process. This configuration ensures consistent, reproducible, and sequential exposure of SPE surfaces to the plasma arc, mimicking the automation used in commercial SPE production. The distance between the coil tip and the electrode surface (plasma power) and the exposure time were optimized to achieve uniform surface treatment. The effects of the robotic air-plasma treatment were systematically characterized using electrochemical techniques, atomic force microscopy (AFM), scanning electron microscopy (SEM), and Raman spectroscopy. The plasma-treated SPEs were subsequently applied to the detection of picric acid in forensic samples. This robotic platform offers a cost-effective alternative to existing plasma treatment systems, minimizing human intervention while ensuring consistent, high-quality surface activation.

## Experimental Section

More information on this topic is provided in Section S1.

### Construction of Robotic Plasma Treatment System

A custom-designed robotic platform was developed to mechanize atmospheric plasma treatment of SPE surfaces, demonstrating its potential for scalable and reproducible surface cleaning, activation, and modification. Figure 1 presents a schematic representation of the proposed treatment protocol applied to the SPE surfaces. A DC8–32 V Music Tesla Arc Plasma Speaker, originally designed for science education (Figure 1C), was adapted to serve as the plasma source in the robotic system. This educational kit integrates both the high-voltage plasma generation circuit and the arc-emitting tip responsible for surface treatment. In its standard configuration, the plasma discharge is modulated by the input audio signal.

**Figure 1:**
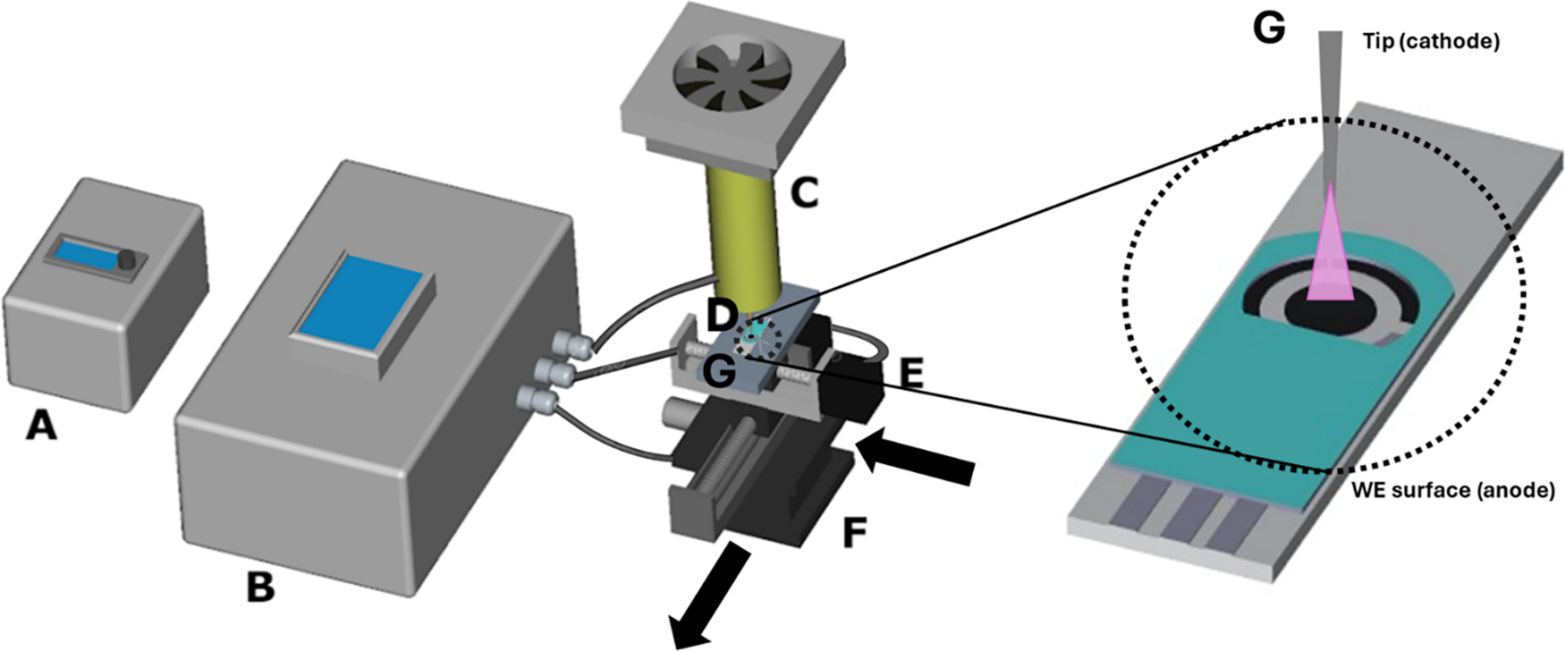
Schematic representation of the laboratory-built robotic plasma treatment system. (A) Plasma power control unit; (B) control unit with graphical interface; (C) atmospheric plasma generator; (D) electrode surface region exposed to plasma treatment (electrode positioning platform); (E, F) CNC motion system for *X*- and *Y*-axes, respectively; and (G) illustration of the plasma effect on the C-SPE surface.

The plasma generation system was powered by a 48 V/5A supply, delivering the necessary energy to sustain the high-voltage discharge. To ensure operational stability and precise control, a regulated XY6020L power supply was used to finely adjust the voltage and current delivered to the plasma generator, enabling consistent and reproducible plasma generation. To robotically automate the treatment process and ensure uniform exposure of the electrode surfaces, a custom-designed motion platform was integrated into the system.

This motion platform (5 cm × 10 cm) was fabricated using additive manufacturing technology with a GTMAX A1 3D printer (GTMAX3D, Campinas, Brazil) and acrylonitrile butadiene styrene (ABS) filament (GTMAX3D, Campinas, Brazil), defining the system’s effective working area (Figure 1D). Its dimensions allow for the simultaneous treatment of multiple small-scale electrodes, enhancing the throughput and overall processing efficiency. The platform is mounted on two orthogonally aligned NEMA 11 CNC linear stages: one with a 50 mm travel range and 2 mm lead screw pitch for controlled displacement along the *Y*-axis (Figure 1E) and another with a 100 mm travel range and 2 mm pitch for movement along the *X*-axis (Figure 1F). This configuration enables precise programmable motion across the treatment plane. The plasma generator is suspended above the platform at a defined vertical distance (*Z*-axis) using an adjustable universal support that ensured consistent alignment and height. To further enhance control over the plasma–surface interaction, a manual micrometer-adjustment stage was integrated, allowing fine-tuning of the gap between the plasma needle and the electrode surface with high precision. This level of control is essential for optimizing the uniformity and effectiveness of plasma treatment. Further details regarding the software development, firmware used, photographs of the robotic system components (Figure S1), and a detailed breakdown of material costs (Table S1) are provided in Section S1.4 (Robotic Plasma System).

The motion system (*XY*-axes) and plasma generation commands are controlled by an electronic unit built around the BIGTREETECH SKR MINI E3 V2.0 32-bit controller board. A schematic illustration of this module is shown in Figure 1A. This board controls the stepper motors for *X* and *Y*-axis motion and manages the on/off switching of plasma generation. Although originally designed for 3D printer control, SKR MINI E3 V2.0 was successfully repurposed to operate the plasma actuation system. To ensure compatibility with the plasma generator and maintain stable operation, specific hardware and firmware modifications were also implemented (Figure S2).

### Plasma Treatment of the SPE Surface

Commercially available carbon SPEs were subjected to plasma treatment using the proposed lab-built robotic platform with the aim of enhancing their electrochemical performance. During the plasma surface activation process, C-SPEs are positioned on the platform (Figure 1D), and the operator uses the micrometric adjustment stage to precisely control the distance between the plasma needle and the electrode surface. The touchscreen interface (Figure 1B) enables the user to define the treatment area by designing a project that specifies the regions to be activated. It also allows the configuration of key treatment parameters that may influence the effect of plasma generation on the electrode surface, potentially affecting the electrochemical behavior of the treated electrodes, such as the spacing between successive plasma passes (adjustable with 0.1 mm precision) and the platform’s movement speed (which can be set from 50 to 400 mm min^–1^). Additionally, the interface supports the creation of various treatment geometries, including elliptical and rectangular patterns. Once the configuration is complete, the Raspberry Pi transmits G-code commands to the controller board, which synchronizes the *X*- and *Y*-axes motions and triggers plasma discharge along the predefined trajectory.

Plasma generation is initiated by applying a high voltage between the plasma generator’s tip (anode) and the surface of the C-SPE (cathode), which ionizes the surrounding atmospheric gases. As the voltage increases, a strong electric field is established, causing dielectric breakdown of the surrounding gas and forming a conductive plasma channel through which the current flows (Figure 1G). This process generates the visible luminous emission characteristic of arc-discharge plasma. A dielectric barrier at the generator tip stabilizes the discharge, enabling localized and efficient plasma treatment. The treatment conditions were systematically optimized in a stepwise manner. The initial parameter values were selected within the operational limits of the robotic platform, based on arbitrary choices, as all tested settings resulted in improved electrochemical performance. The goal of the univariate evaluation was to identify whether specific parameters could yield superior results for the type of electrode surface studied. This analysis defined the optimal conditions, which were applied during treatment, including a needle-to-surface distance of 1.5 mm, plasma power of 12.9 W, line spacing of 0.2 mm, and movement speed of 250 mm·min^–1^. Once established, these conditions were programmed in the robotic platform, enabling precise, reproducible, and rapid plasma surface treatment (approximately 136 s per cm^2^ or 16 s per working electrode of an SPE device). The video available in the Supporting Information demonstrates the lab-built robotic plasma system activating five working electrodes from distinct SPE devices.

## Results and Discussion

### Optimization of SPE Surface Activation Using the Lab-Built Robotic Air-Plasma System

Initially, the performance of the lab-built robotic atmospheric air-plasma system was evaluated for the surface activation of commercially available C-SPEs. To identify the optimal activation conditions, the electrochemical response of each C-SPE was evaluated before and after plasma treatment by cyclic voltammetry (CV) using a 1.0 mmol L^–1^ [Fe­(CN)_6_]^3–/4–^ redox probe in 0.10 mol L^–1^ KCl supporting electrolyte. Treatment parameters were systematically varied according to a univariate approach. The voltammograms corresponding to the univariate optimization of parameters such as distance from the electrode surface (plasma power decreases with increasing distance), spacing between plasma lines, and treatment rate (mm min^–1^) are shown in Figures S3, S4, and S5, respectively. To investigate the effect of each parameter, the increase in anodic peak current (*I*_pa_) and the improvement in redox reversibility, measured by a decrease in peak-to-peak separation (Δ*E*_p_), were evaluated relative to both untreated (control) and plasma-treated surfaces under each condition. The values obtained for each parameter evaluated are presented in Table S2. Further discussion and details regarding the selection of parameters are provided in Section S2, page S12.

Intermediate conditions were selected in future experiments as follows: distance = 1.5 mm; line spacing = 0.2 mm; and scan rate = 250 mm min^–1^. Under these conditions, the interelectrode precision of the proposed surface activation protocol was evaluated by CV in the presence of 1.0 mmol L^–1^ [Fe­(CN)_6_]^3–/4–^ in 0.10 mol L^–1^ KCl solution, using C-SPEs before and after robotic air-plasma treatment. For this purpose, three C-SPEs from each of the three distinct batches were employed (*n* = 3 per batch). Figure 2 presents the corresponding voltammetric responses, while Table 1 summarizes the mean values of *I*pa and Δ*E*_p_, along with their respective relative standard deviations (RSDs), calculated for each group of three SPEs per batch, before and after plasma treatment. A substantial increase in peak current intensities (7.5 ± 1.1 to 14.0 ± 1.7 μA; *n* = 3) for both oxidation and reduction processes was observed on the plasma-treated surfaces, accompanied by enhanced electrochemical reversibility (Δ*E*_p_ of 287 ± 43 mV to 81 ± 6 mV; *n* = 3), as evidenced by the improved voltammetric profiles across all analyzed batches. Furthermore, as shown in Figure 2 and Table 1, the variation in both *I*_pa_ and Δ*E*_p_ is smaller between the plasma-treated SPEs compared to the untreated ones, indicating that plasma treatment enhances the interelectrode reproducibility, a critical factor for the consistent performance of disposable electrodes.

**Figure 2:**
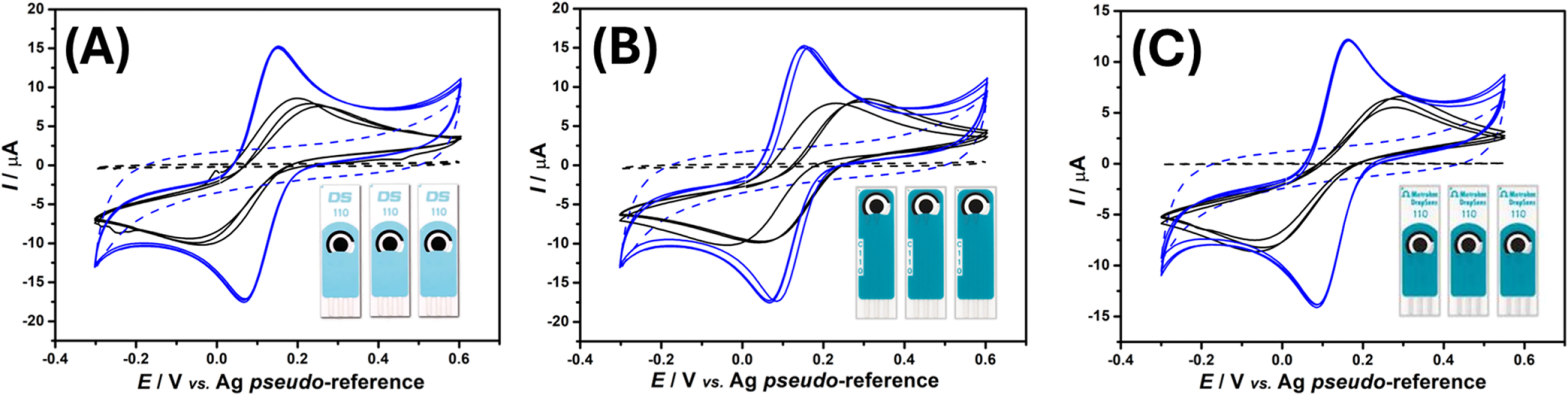
Cyclic voltammetric responses before (black lines) and after (blue lines) plasma treatment for C-SPEs from batch 1 (A), batch 2 (B), and batch 3 (C), recorded in the presence of 1.0 mmol L^–1^ [Fe­(CN)_6_]^3–^/^4–^ in 0.1 mol L^–1^ KCl solution, using three electrodes from each batch (*n* = 3). Each set of voltammograms (untreated and treated) was obtained from three individual electrodes from the same batch. The dashed lines represent blank signals. CV conditions: Scan rate = 50 mV s^–1^; step potential = 5 mV.

**Table 1:** Δ*E*_p_ and *I*_pa_ Values for C-SPEs from Three Different Batches (*n* = 3 per Batch) under Optimized Plasma Treatment Conditions^a^

	untreated SPE				plasma-treated SPE			
C-SPEs batch	Δ*E*_p_ (mV)	*I*_pa_ (μA)	RSD (%, Δ*E*_p_)	RSD (%, *I*_pa_)	Δ*E*_p_ (mV)	*I*_pa_ (μA)	RSD (%, Δ*E*_p_)	RSD (%, *I*_pa_)
1	274 ± 16	8.0 ± 0.6	5.8	7.5	87 ± 3	15.2 ± 0.2	3.5	1.3
2	252 ± 10	8.2 ± 0.3	4.0	3.7	81 ± 3	15.1 ± 0.1	3.7	0.7
3	335 ± 27	6.2 ± 0.6	8.1	9.7	76 ± 1	12.1 ± 0.1	1.3	0.8

a RSD values are also reported for untreated and treated surfaces, calculated from three electrodes per batch. Data were obtained from voltammetric responses measured in the presence of 1.0 mmol L^–1^ [Fe­(CN)_6_]^3–^/^4–^ in a 0.1 mol L^–1^ KCl solution.

### Interelectrode Reproducibility: Comparison between Manual and Robotic Plasma Activation

Recently, Siqueira et al.^19^ and Marra et al.^23^ explored the application of a hand-held plasma pen, originally designed for dermatological and esthetic purposes, for the efficient treatment of carbon-based surfaces, applying a power of 3 W for 2 min. The operating principle of the plasma pen is similar to that of the robotic system proposed herein, relying on an electric arc generated between the tip of the plasma pen and the target surface to ionize atmospheric gases. In this approach, the pen is manually guided across the electrode surface to perform the activation protocol. Despite their effectiveness, manual methods often suffer from limited reproducibility and present challenges in scalability, making them less suitable for large-scale production of electrochemical sensors such as SPEs. To compare the interelectrode precision between plasma treatments performed by the pen and the robotic system, CV measurements were carried out in the presence of 1.0 mmol L^–1^ [Fe­(CN)_6_]^3–/4–^ in 0.1 mol L^–1^ KCl solution, using three distinct C-SPEs (*n* = 3) before and after treatment with both systems (Figure S6).

The plasma pen activation procedure was carried out by three different analysts from our research group, each treating a separate electrode under the same previously optimized plasma pen conditions, as reported in the literature.^19^ Table S3 presents the *I*_pa_ and Δ*E*_p_ values and the corresponding RSDs for each parameter. Figure S6A shows that the plasma pen treatment, even when performed by different analysts, improved the voltammetric profile of the redox probe, as evidenced by increased current intensities and a reduced peak-to-peak separation (Δ*E*_p_) compared to untreated surfaces, yielding results comparable to those obtained with the robotic surface activation system (Figure S6B and Table 1). Nonetheless, regarding interelectrode precision, C-SPEs activated by the robotic plasma system showed significantly more consistent performance, with an RSD of 0.7% for *I*_pa_ and 1.6% for Δ*E*_p_ (Table 1, batch 3). In contrast, C-SPEs from the same batch treated with the atmospheric air-plasma pen exhibited greater variability, with an RSD of 10.4% for Ipa and 3.3% for Δ*E*_p_ (Table S3). These findings suggest that the robotic plasma method not only improves electrochemical performance but also significantly enhances reproducibility by minimizing human error, which is crucial for the large-scale production of disposable electrodes.

### Extension of the Robotic Plasma Treatment to Diverse Electrode Materials and Architectures

Furthermore, to assess the feasibility and effectiveness of robotic plasma treatment on surfaces composed of different electrode materials, commercial SPEs fabricated from carbon, gold, and platinum, stored under ambient conditions for approximately 10 years, were subjected to robotic plasma activation under optimized treatment conditions. These electrodes were initially provided as complementary samples and remained unused in our laboratory. Although they were not originally designated for this study, their extended storage period presented a valuable opportunity to assess whether plasma treatment could effectively restore electrochemical activity despite the potential formation of passivation layers and the progressive accumulation of impurities over time. A significant enhancement in voltammetric performance was observed for all types of SPEs evaluated following the robotic plasma treatment. Figure S7 presents the cyclic voltammetric responses obtained for commercial gold AT SPEs before and after robotic plasma treatment (*n* = 3). As shown, a substantial increase in peak current intensity for both oxidation and reduction processes was observed following the activation protocol, along with enhanced electrochemical reversibility (*I*_pa_ increased from 7.8 ± 4.0 μA to 13.9 ± 0.8 μA; Δ*E*_p_ decreased from 124 ± 29 to 73 ± 4 mV). Figure S8 displays the analogous results for platinum (550) SPEs (*n* = 2) and an increase of *I*_pa_ (from 5.8 ± 0.5 μA to 14.6 ± 2.5 μA), together with a decrease in peak-to-peak separation (Δ*E*_p_ from 208 ± 40 to 108 ± 6 mV), was observed. Figure S9 depicts the results obtained for the gold BT SPE surfaces (*n* = 2), and, consistent with the previously studied materials, a pronounced enhancement in the voltammetric profile was observed following robotic plasma treatment (*I*_pa_ increased from 3.7 ± 1.4 μA to 14.1 ± 0.2 μA; Δ*E*_p_ decreased from 649 ± 85 mV to 76 ± 1 mV). Consistent improvements were also observed for carbon-based SPEs (*n* = 3) under identical treatment conditions (*I*pa increased from 3.4 ± 0.4 μA to 6.6 ± 0.6 μA; Δ*E*_p_ decreased from 296 ± 34 to 76 ± 1 mV) (Figure S10). These results highlight the potential of the robotic plasma method for reactivating SPE surfaces that have undergone long-term storage.

Subsequently, a 3D-printed working electrode fabricated as described in the Experimental Section, using a filament containing carbon black and polylactic acid (CB/PLA) was also employed to investigate the effectiveness of the robotic plasma treatment in enhancing the electrochemical behavior of alternative electrode architectures that can be fabricated on a large scale and may present reproducibility challenges or require additional surface activation. The plasma treatment was performed using the same optimized conditions established for SPEs in this work, and CV measurements were carried out in 1.0 mmol L^–1^ [Fe­(CN)_6_]^3–/4–^ with 0.1 mol L^–1^ KCl. As shown in Figure S11, the untreated 3D-printed CB/PLA electrode exhibited no evident redox peaks, confirming the need for surface activation prior to use. In contrast, after plasma treatment, a substantial improvement in the electrochemical response was observed with well-defined and intense redox peaks and satisfactory peak separation. These results demonstrate that the robotic plasma system can be effectively applied to different carbon-based surfaces and electrode configurations. This finding extends the applicability of the developed platform beyond commercial SPEs and highlights its strong potential for future use in the scalable and reproducible activation of diverse electrochemical devices.

Next, a comprehensive analysis was conducted to assess the effects of plasma treatment on C-SPEs surfaces using Raman spectroscopy, atomic force microscopy (AFM), scanning electron microscopy (SEM), and electrochemical measurements. These characterizations were performed both before and after plasma activation to enable a direct comparison.

### Morphological and Spectroscopic Characterization

Initially, SEM analyses were conducted to evaluate the morphological alterations of the C-SPE surface following the robotic air-plasma treatment (Figure 3A1,B1). As shown in Figure 3B1, the robotically plasma-treated C-SPE exhibited irregular surface features, characterized by flaking and formation of additional edge-like structures, in clear contrast to the smoother and more homogeneous morphology observed on the untreated electrode (Figure 3A). These observations suggest a significant restructuring of the material into a more complex and roughened surface, likely resulting from the removal of surface impurities and exposure of conductive graphite domains. This behavior aligns with previous findings reported in the literature.^19,23–25^

**Figure 3:**
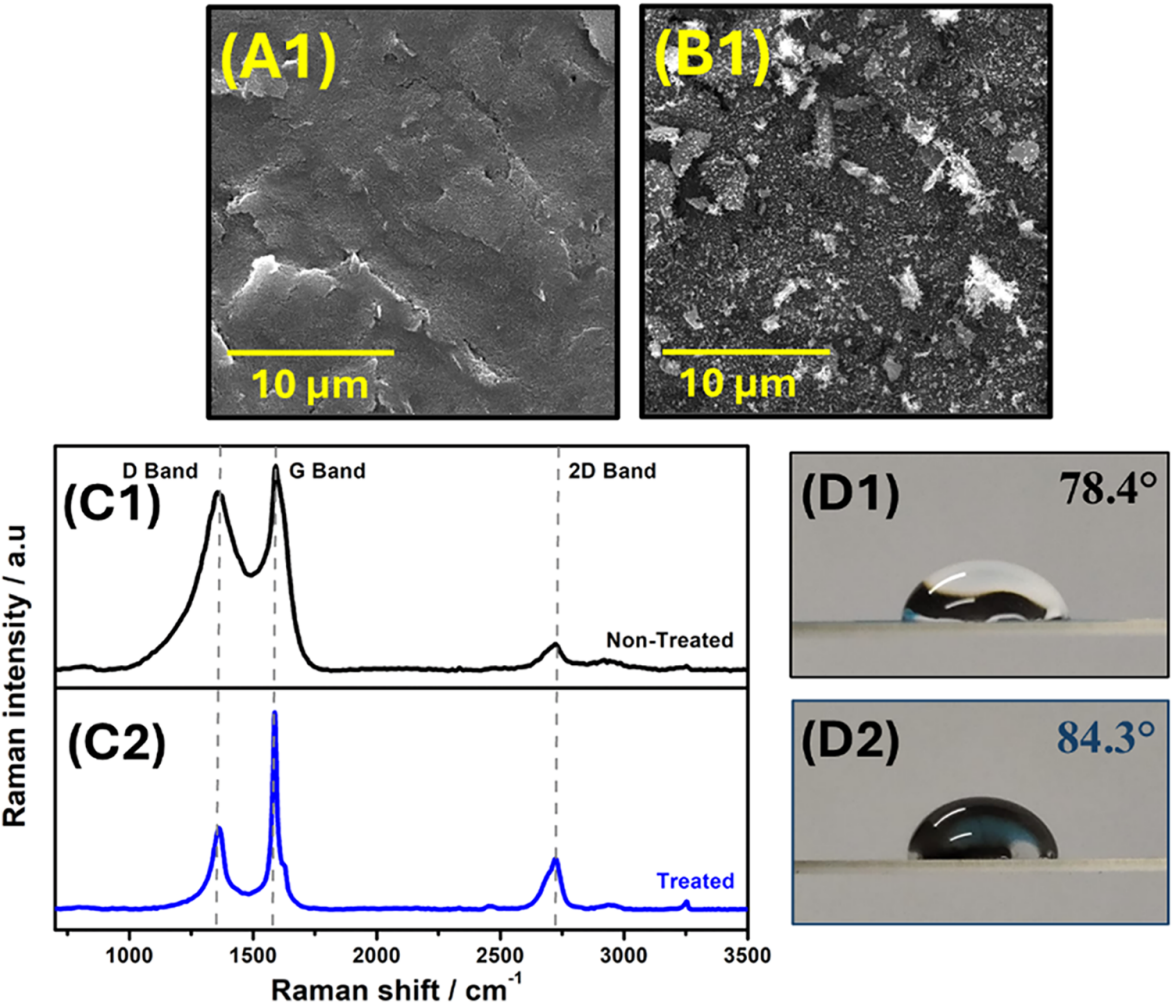
SEM images of the C-SPE surface obtained before (A1) and after (B1) robotic air-plasma treatment. Raman spectra were obtained for the untreated (black line) (C1) and robotic air-plasma-treated (blue line) (C2) C-SPE. (D1, D2) Contact angle measurements were performed on untreated and plasma-treated C-SPE surfaces, respectively.

Next, AFM analysis was employed to further investigate the surface topography of both untreated and robotically plasma-treated C-SPEs, with the corresponding images presented in Figure S12. The AFM images demonstrate that plasma treatment significantly increased the surface roughness compared with the untreated C-SPE. The root-mean-square roughness (RMS) of the untreated C-SPE was measured at 36.7 nm (Figure S12A), indicating a relatively smooth and compact surface morphology. Conversely, the robotically air-plasma-treated C-SPE (Figure S12B) exhibited a substantial increase in surface roughness, evidenced by the development of pronounced topographical features and additional edge-like structures, with the RMS roughness increasing to 165.4 nm. These AFM results are in good agreement with the morphological changes observed in the SEM analysis, further confirming the significant surface restructuring induced by plasma treatment. Analysis of both AFM and SEM images reveals that the air-plasma treatment induced the formation of irregular surface patterns, resulting in a more porous and uneven surface morphology. Such topographical modifications are likely to increase the effective surface area and facilitate analyte access to electroactive sites—factors that are expected to contribute to the improved electrochemical performance of the C-SPEs.

Subsequently, Raman spectroscopy analysis was performed on the C-SPE surfaces before and after the robotic air-plasma treatment (Figure 3C) to evaluate the effects of the proposed surface activation procedure. The analysis aimed to assess the presence of defects and investigate potential changes in structural disorder within the graphitic surface induced by plasma treatment. In Figures 3C1 and C2, the characteristic bands of carbonaceous materials can be observed: D band (∼1355 cm^–1^), G band (∼1587 cm^–1^), and 2D band (∼2723 cm^–1^).^24^ The G band corresponds to the vibrational mode of sp^2^-hybridized carbon atoms within the graphitic structure, while the D band is associated with structural defects and disorder in the graphite layers. The 2D band (∼2723 cm^–1^) provides information about the number of stacked graphene layers, and its intensity can also be affected by structural disorder and defects.^26^ The intensity ratio of the G and D bands (*I*_D_/*I*_G_) is commonly used to obtain more detailed information regarding the degree of crystalline disorder in carbon-based materials.^16,27^

Considering the data obtained from the deconvolution of the Raman spectra, the intensity of the D band slightly decreased after plasma treatment, while the G and 2D bands showed a notable increase in intensity following the proposed modification. The reduced intensity of the D band observed at the plasma-treated surface of the C-SPE suggests a lower density of basal plane defects across its lattice and the increase in the G band indicates that the graphitic structure became more exposed following the proposed activation protocol.^24^ The greater intensity of the G band relative to the 2D band is characteristic of multilayered graphene structures.^28,29^ Thus, the untreated C-SPE showed an *I*_D_/*I*_G_ ratio of 0.95, whereas the plasma-treated electrode exhibited a reduced *I*_D_/*I*_G_ ratio of 0.42. These findings indicate that plasma treatment promotes a more ordered crystalline structure on the electrode surfaces, as evidenced by the lower defect density (decrease in the *I*_D_/*I*_G_ ratio), likely due to the effective removal of binder residues from the electrode surface.^24^

The ratio between the 2D and G bands (*I*_2D_/*I*_G_) provides valuable insights into the classification of graphitic material. The *I*_2D_/*I*_G_ ratio can be used to determine the number of graphene layers present in a structural material.^30^ Ratios below 1 are indicative of multilayer graphene, while ratios above 1 typically correspond to monolayer graphene.^31^ *I*_2D_/*I*_G_ values of 0.14 and 0.28 were obtained for untreated and air-plasma-treated C-SPE, respectively. These values indicate that both bare and treated C-SPEs are composed of multilayer graphene,^29^ although the C-SPE treated with atmospheric air plasma exhibits a slightly lower number of graphene layers. This behavior is attributed to the nanostructuring effect induced by plasma ablation, which removes disordered carbon and partially exfoliates the graphitic structure. Crystalline graphite typically exhibits a well-defined 2D band, a feature associated with its high degree of structural order. Disruptions or increases in structural disorders tend to degrade the definition and intensity of this band. The clear presence of the 2D band in the plasma-treated C-SPEs, along with the observed increase in its intensity and sharpness, indicates an improvement in the crystalline quality of the electrode surface.

Given that the treatment enhanced the definition of the graphitic peaks, it can be inferred that the plasma process promoted a greater ordering or more exposure of the crystalline structure.^24^ Based on these observations, it can be inferred that the plasma treatment removed oxygen-containing functional groups from the surface, consistent with the elimination of the polymer binder, and increased the relative concentration of sp^2^ carbon due to the exposure of the conductive graphitic sites. As previously reported, laser^24^ and plasma^32^ treatments of carbon SPE surfaces can enhance graphite crystallinity and improve electron-transfer rates, likely due to the removal of the polymeric binder.

The water contact angle (θ), defined as the angle between the tangent to a hemispherical droplet at the point of contact and a line parallel to the WE surface, increases as the surface hydrophobicity increases.^33^ Additionally, measuring this parameter provides valuable insights into the surface termination both before and after plasma treatment.^34^ Thus, water contact angle measurements were performed to evaluate whether the plasma treatment affected the wettability properties of the C-SPE surface. As can be observed in Figures 3D1 and D2, the untreated C-SPE exhibits a contact angle of 78.4°, whereas the plasma-treated surface shows an increased value of 84.3°, indicating enhanced hydrophobicity. This behavior is consistent with the Raman spectroscopy results, which suggested that the plasma treatment did not introduce oxygen-containing functional groups but rather removed polymeric binders, impurities, and other nonconductive residues from the surface. The removal of these polar contaminants and the increased exposure of the more ordered and conductive sp^2^ carbon domains, which are inherently hydrophobic, likely contributed to the observed increase in the water contact angle.^24,35^

### Electrochemical Characterization

Given that the plasma treatment resulted in an increase in the surface roughness of the C-SPEs (an effect commonly associated with an increased electroactive surface area), as observed by AFM and SEM images, we aimed to estimate this parameter. Thus, we measured the double-layer capacitance (*C*_dl_) of both untreated and robotically plasma-treated C-SPEs, as described in the Experimental Section. Since *C*_dl_ is directly proportional to the electroactive surface area,^36^ it serves as an indirect measure of the surface activation induced by the treatment. A higher Cdl value corresponds to a greater electroactive surface area, as defined by eq S1 (experimental section in the Supporting Information). The *C*_dl_ value obtained for the untreated C-SPE (Figure 4A) was 73.29 μF cm^–2^, while the value measured after plasma treatment was 269.53 μF cm^–2^ (corresponding to a 3.7-fold increase). Then, the *A*_ele_ was estimated for both the untreated and plasma-treated C-SPEs, using their geometric area and the specific capacitance (*C*_s_, theoretical value of 110 μF cm^–2^ for C-SPE),^37^ employing eq S1, as described in the experimental section in the Supporting Information. The estimated *A*_ele_ value for the untreated surface was 0.07 cm^2^, whereas for C-SPE subjected to plasma treatment, it increased to 0.27 cm^2^. These results agreed with the morphological analyses (AFM and SEM images), confirming the increase in surface roughness. This finding is consistent with the effect of plasma treatment, which exposes more conductive graphitic sites and enhances the electroactive area.

**Figure 4:**
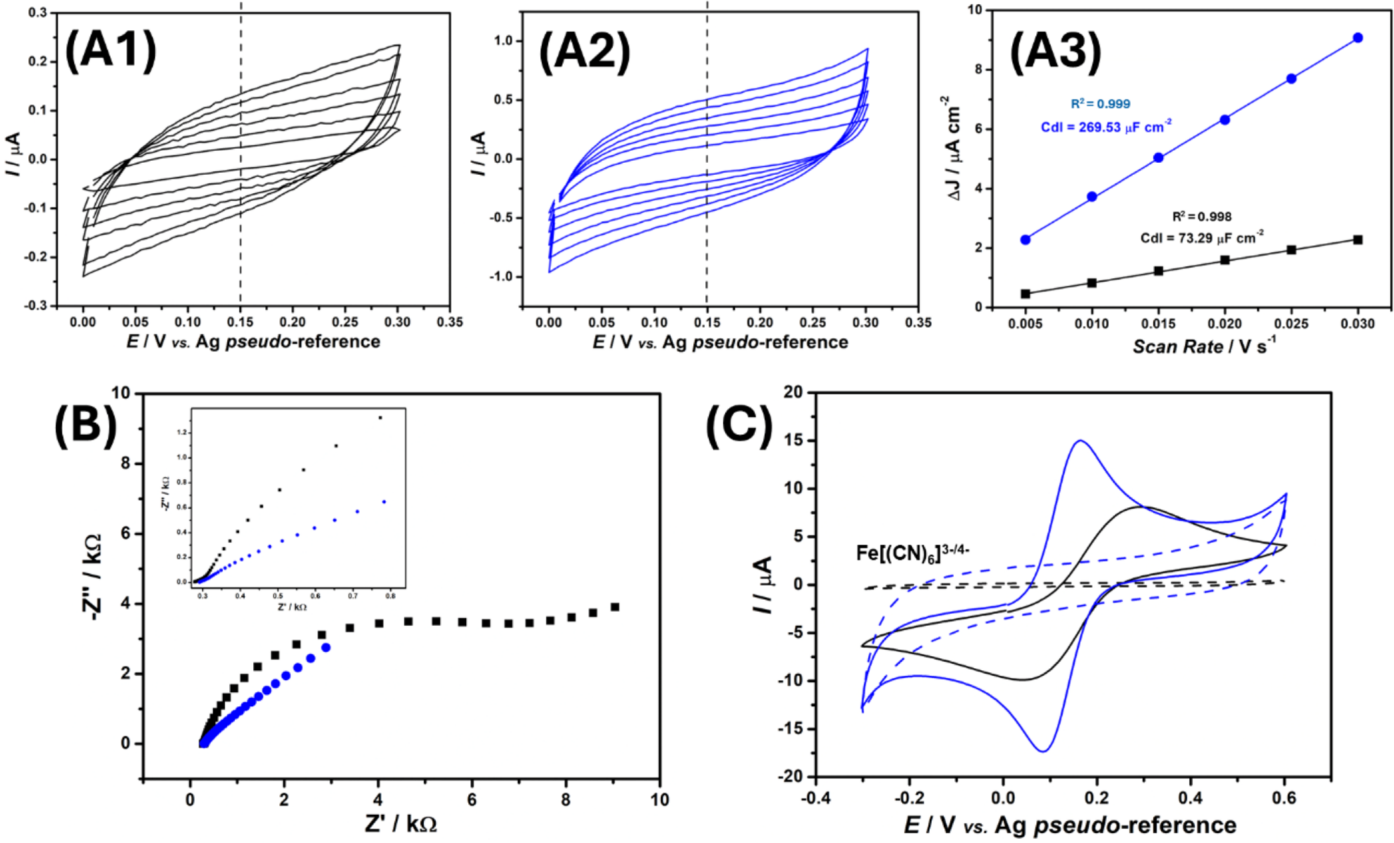
Cyclic voltammograms were recorded at scan rates ranging from 5 to 30 mV s^–1^ for the untreated (A1) and robotic plasma-treated (A2) C-SPE. The cyclic voltammetric measurements were carried out in 0.1 mol L^–1^ KCl solution, within the potential window of 0.0 to +0.3 V (*vs* Ag pseudo-reference electrode). (A3) Capacitance data for the untreated (black line) and plasma-treated (blue line) C-SPEs, represented as plots of Δ*J* (peak current measured at +0.15 V vs. Ag pseudo-reference) versus the CV scan rate for each surface. Step potential = 5 mV. (B) Nyquist plots from EIS measurements recorded in the presence of 1.0 mmol L^–1^ [Fe­(CN)_6_]^3–/4–^ in 0.1 mol L^–1^ KCl solution, applying +0.23 V (*vs* Ag|AgCl|KCl_(sat.)_) for untreated C-SPE (black line) and +0.18 V (*vs* Ag|AgCl|KCl_(sat.)_) for the robotically plasma-treated C-SPE (blue lines). Frequency range: 50.000–0.1 Hz; amplitude: 10 mV. (C) Corresponding CV responses for untreated (black lines) and robotic plasma-treated (blue lines) C-SPE, recorded in the presence of 1.0 mmol L^–1^ [Fe­(CN)_6_]^3–/4–^ in 0.1 mol L^–1^ KCl. Scan rate: 50 mV s^–1^; step potential: 5 mV.

Afterward, electrochemical impedance spectroscopy (EIS) measurements were performed on the untreated and robotically plasma-treated C-SPE in the presence of 1.0 mmol L^–1^ [Fe­(CN)_6_]^3–/4–^, as shown in Figure 4B. From the Nyquist plots, recorded at the half-wave potential (*E*_1/2_) of the [Fe­(CN)_6_]^3–/4–^ redox probe on each surface, and analyzed using the corresponding Randles equivalent circuit, the charge-transfer resistance (*R*_ct_) values were determined to be 7.10 ± 0.14 kΩ for the untreated C-SPE and 0.45 ± 0.05 kΩ for plasma-treated C-SPE. Subsequently, to estimate the heterogeneous electron-transfer rate constant (*k* ^0^) for both untreated and plasma-treated C-SPEs surfaces, the *R*_ct_ data extracted from the Nyquist plots and fitted Randles equivalent circuits were used, following the Sluyters’s method,^38,39^ as shown in the equation below

$$k^{0}=\frac{\mathrm{RT}}{F^{2}A_{\mathrm{ele}}C\times R_{\mathrm{ct}}}$$

The calculations were performed assuming *R* = 8.314 J mol^–1^ K^–1^, *T* = 298 K, and *F* (Faraday’s constant) = 96,485 C mol^–1^. The estimated *k* ^0^ values were 0.513 × 10^–3^ cm s^–1^ for the untreated C-SPE and 2.194 × 10^–3^ cm s^–1^ for the plasma-treated C-SPE, suggesting an electron transfer rate around 4-fold higher on plasma-treated C-SPE.

To further investigate the electron-transfer kinetics at the electrode surface, both untreated and plasma-treated C-SPEs were analyzed by cyclic voltammetry in the presence of 1.0 mmol L^–1^ [Ru­(NH_3_)_6_]^2+/3+^ in 0.1 mol L^–1^ KCl solution, while the scan rate (*v*) was varied from 10 to 200 mV s^–1^ (data not shown). Through eqs S2 and S3 (experimental section in the Supporting Information), *k* ^0^ was determined directly from the slope of the plot of Ψ vs [πDnF*v*/*RT*]^−1/2^.^38,40^ As anticipated, the robotically plasma-treated C-SPE exhibited a higher *k* ^0^ value (4.29 ± 0.95 ×10^–3^ cm s^–1^) relative to the untreated surface (0.82 ± 0.07 × 10^–3^ cm s^–1^), suggesting improved heterogeneous electron-transfer kinetics (∼5-fold increase). Notably, despite *k* ^0^ being estimated using two different methodologies (Sluyters’s and Nicholson’s methods), both approaches yielded consistent values within the same order of magnitude, reinforcing the observed enhancement in *k* ^0^ following the plasma treatment. The changes in *R*_ct_ and *k* ^0^ values observed for the C-SPEs after plasma treatment can be attributed to the removal of surface impurities, which expose additional conductive sites. Moreover, the treatment likely induces structural modifications that improved analyte–electrode interactions, further enhancing electron-transfer kinetics.

These findings emphasize the significant role of plasma treatment in enhancing the electrochemical performance of C-SPEs by both increasing the electroactive area and modifying the surface properties to favor electron transfer at the interface. An overview of the parameters obtained from the electrochemical characterization section is presented in Table 2.

**Table 2:** Electrochemical Parameters, Including Peak-to-Peak Separation (Δ*E*_p_), Heterogeneous Electron-Transfer Rate Constant (*k* ^0^) Obtained by Two Different Methodologies (^a^Sluyters’s and ^b^Nicholson’s methods), Electroactive Surface Area (*A*_ele_), and Charge-Transfer Resistance (*R*_ct_) for the C-SPE before and after Robotic Air-Plasma Treatment

	C-SPE	
parameters	untreated	treated
^a^Δ*E*_p_/mV	252 ± 10	81 ± 3
^a^ *I*_pa_/μA	8.2 ± 0.3	15.1 ± 0.2
^a^ *k* ^0a^/× 10^–3^ cm s^–1^	0.513 ± 0.09	2.194 ± 0.02
^b^ *k* ^0b^/× 10^–3^ cm s^–1^	0.82 ± 0.07	4.29 ± 0.95
*A*_ele_/cm^2^	0.073 ± 0.012	0.270 ± 0.037
^a^ *R*_ct_^a^/kΩ	7.10 ± 0.14	0.45 ± 0.05

a The analyses were performed using 1.0 mmol L^–1^ [Fe­(CN)_6_]^3–/4–^ in 0.1 mol L^–1^ KCl solution.

b The analyses were conducted using 1.0 mmol L^–1^ [Ru­(NH_3_)_6_]^2+/3+^ in 0.1 mol L^–1^ KCl solution. Scan rate in both experiments: 50 mV s^–1^.

Finally, to comprehensively assess the effects of the proposed plasma treatment and explore its impacts on the voltammetric response of various electroactive species, the electrochemical behavior of the plasma-treated C-SPE was investigated by recording voltammetric responses for several organic compounds, including dopamine (DOP), ascorbic acid (AA), tyrosine (TRY), uric acid (UA), nitrite (NIT), and picric acid (PA), all at 1.0 mmol L^–1^, as illustrated in Figure S13. The plasma treatment notably affected the electrochemical behavior of all of the investigated analytes. For DOP, AA, and UA, a clear shift in the oxidation potentials toward less positive values (indicating a reduction in overpotentials) was observed, accompanied by a significant increase in the peak current intensities. This suggests that the treatment facilitated electron-transfer processes, likely due to improved exposure of conductive sites and a reduction in fouling effects.

Notably, the plasma treatment also resulted in a substantial improvement in the electrochemical reversibility of the electrochemical processes of DOP (Figure S13A), as evidenced by the significant decrease in peak-to-peak separation (Δ*E*_p_ from 299 to 65 mV). In addition, these findings indicate not only improved charge-transfer kinetics but also a more favorable interaction between the species and the treated electrode surface. The observed catalytic voltammetric behavior can be directly attributed to morphological modifications induced by plasma treatment, likely associated with the removal of binders and/or impurities from the surface of the commercial SPEs, as previously reported by other authors using plasma treatment on comparable carbon-based electrodes.^32^ These changes enhance the electrode’s ability to promote electron transfer, particularly for analytes that have greater affinity for or interact more strongly with the plasma-treated surface, where the presence of newly exposed active sites and altered surface chemistry play critical roles.^41^ Furthermore, TYR and NIT exhibited higher oxidation currents after plasma treatment, along with a slight reduction in overpotentials, highlighting the enhanced electron-transfer kinetics even for these more challenging oxidation processes.

The electrochemical behavior of PA has been previously reported in the literature; however, depending on the experimental conditions, between one and four reduction signals can be observed, which are attributed to the reduction of the nitro groups.^42,43^ Regarding PA (Figure S13F), the untreated C-SPE exhibited a weak electrochemical response in acidic medium, characterized by three poorly defined reduction peaks at approximately −0.3, −0.4, and −0.6 V (vs Ag *pseudo*-reference electrode), and a low-intensity oxidation peak near 0.0 V (vs Ag *pseudo*-reference electrode).^44^ In contrast, the plasma-treated C-SPE surface showed an improved voltammetric response with better-defined peaks and increased current intensities compared to the untreated surface. Moreover, the cathodic processes of PA shifted to less negative potentials following plasma treatment, indicating a reduction in the overpotential required for these processes.

The effect induced by the plasma treatment enabled the cathodic processes to be observed at approximately −0.1 V (Red 1), −0.2 V (Red 2), −0.4 V (Red 3), and −0.6 V (Red 4) (*vs* Ag *pseudo*-reference electrode), all accompanied by increased peak current intensities. In contrast, for the anodic faradaic process (Ox1) observed around 0.0 V (*vs* Ag *pseudo*-reference electrode), a considerable increase in peak current was observed; however, the morphological changes resulting from the electrode surface did not promote a catalytic effect for this specific process.

These results confirm that plasma treatment substantially improves the electrochemical performance of C-SPEs, facilitating faster and more efficient redox processes involving diverse electroactive species.

### Electrochemical Behavior of PA at the Robotically Plasma-Treated C-SPE

As a proof of concept, the robotically plasma-treated C-SPE was employed for the determination of PA in explosive samples. First, the effect of pH on the reduction processes of PA was investigated by SWV using the air-plasma-treated C-SPE, in the presence of 30 μmol L^–1^ PA in a BR buffer solution (pH values ranging from 2 to 12). Figure S14A shows that although the peak related to the reduction of PA was observed under all conditions, not all electrochemical processes could be clearly distinguished throughout the entire pH range. The Red 4 process was not considered in this evaluation as its signal overlapped with the oxygen-reduction peak in most of the conditions studied (no oxygen removal was preferred due to onsite applications). Furthermore, since under most of the tested conditions the peak potential of Red 1 was either very close to or overlapped with the reduction signal observed in the blank, this process was also not further investigated. At lower pH values, the reduction signals corresponding to Red 2 and Red 3 are well-separated and clearly defined. However, as the pH increases, these faradaic processes gradually begin to overlap. From an analytical perspective, this loss of signal differentiation is significant as the ability to distinguish between different electrochemical processes in practical applications enables the selective detection and quantification of PA in real samples, which may contain potential interfering species. Therefore, the differentiation of these electrochemical processes is critically important for analytical application.

Additionally, as the pH increased, the reduction processes corresponding to Red 1, Red 2, and Red 3 shifted to a more negative potential (Figure S14B1). This behavior is attributed to the deprotonation of PA in more basic media, leading to the formation of a negatively charged oxygen atom, which is strongly stabilized by the nitro groups.^42^ Consequently, the reduction process becomes more difficult, requiring a higher overpotential to occur at the electrode surface under higher pH conditions. Consistent with this behavior, the Red 2 and Red 3 processes exhibited a linear relationship between *E*_p_ and pH, with slope values of 57 and 44 mV at pH^–1^, respectively. These values are relatively close to the theoretical Nernstian slope of 59 mV pH^–1^, suggesting that an equal number of protons and electrons are involved in these electrochemical processes. Furthermore, this experiment allowed for the identification of the pH conditions in which both Red 2 and Red 3 are observable and exhibit the most favorable electrochemical behavior (Figure S14B2).

Additionally, both reduction processes of PA showed better peak separation, smaller half-peak widths, satisfactory peak currents with lower standard deviations, and overall more well-defined peaks at pH 2.0. Based on these findings, the influence of different supporting electrolytes on the electrochemical response of PA was further investigated. Among the tested conditions, 0.1 mol L^–1^ HCl provided the highest peak intensities for both Red 2 and Red 3 processes, as shown in Figure S14C, and was therefore selected as the supporting electrolyte for subsequent studies.

The electrochemical profile of PA, obtained by CV under optimized pH and supporting electrolyte conditions, is shown in Figure S14D, providing a broader overview of the analyte’s faradaic processes. As observed, during the first scan, starting from −0.1 V toward more cathodic potentials, three reduction processes are detected (Red 2, Red 3, and Red 4). These correspond to the sequential reduction of nitro groups to hydroxylamine derivatives, which are subsequently reduced to amine derivatives in a second reaction step.^45–47^ On the reverse scan (toward anodic potentials), a reversible oxidation process (Ox1) of PA is observed at approximately +0.1 V (*vs* Ag *pseudo*-reference electrode), which is attributed to the oxidation of a hydroxylamine derivative back to a nitro group. In the subsequent scan, following the oxidation of the hydroxylamine derivative (Ox1), the reduction process Red 1, corresponding to the reduction of the nitro group formed in the previous oxidation step, becomes evident. Thus, based on the data obtained in this work, together with previously reported literature findings, we propose a possible redox route for PA on the plasma-treated C-SPE surface, as illustrated in Scheme S1.

In the next step, the SWV parameters (step potential (Δ*E*_s_), modulation amplitude (*a*), and frequency (*f*)) were systematically optimized in triplicate (*n* = 3) using univariate tests in order to improve the analytical performance. The optimal conditions were selected based on the peak current intensity (*I*_p_), peak half-width (*W*_1/2_), lower standard deviation, and suitable differentiation between the Red 2 and Red 3 reduction processes of PA (Figure S15). It was observed that the best electrochemical response for the reduction processes was obtained under the following conditions: *f* = 10 s^–1^; *a* = 70 mV; and Δ*E*_s_ = 7 mV. Thus, they were selected for subsequent studies.

Following the optimized experimental conditions, analytical calibration curves were constructed with increasing concentrations of PA using both untreated (Figure 5A) and plasma-treated (Figure 5B) C-SPEs, with a focus on the Red 2 and Red 3 reduction processes of the analyte. Regarding the untreated C-SPE surface, linear ranges from 1.0 to 50.0 μmol L^–1^ for Red 2 and from 10.0 to 50.0 μmol L^–1^ for Red 3 were obtained. In contrast, the plasma-treated C-SPE displayed significantly enhanced analytical performance (Figure 5C), exhibiting linear ranges from 0.5 to 50.0 μmol L^–1^ for both Red 2 and Red 3 (Figure 5D1 and D2, respectively), demonstrating a substantial increase in detectability. The limit of detection (LOD) and limit of quantification (LOQ) were calculated following the procedure recommended by the International Union of Pure and Applied Chemistry (IUPAC),^48^ where LOD = 3σ/s and LOQ = 10σ/s, with σ representing the standard deviation of the intercept and s denoting the analytical sensitivity (slope) of the calibration curve. Table S4 summarizes the analytical parameters obtained using both the untreated and plasma-treated C-SPEs, including the linear range, LOD, and slope (analytical sensitivity) values.

**Figure 5:**
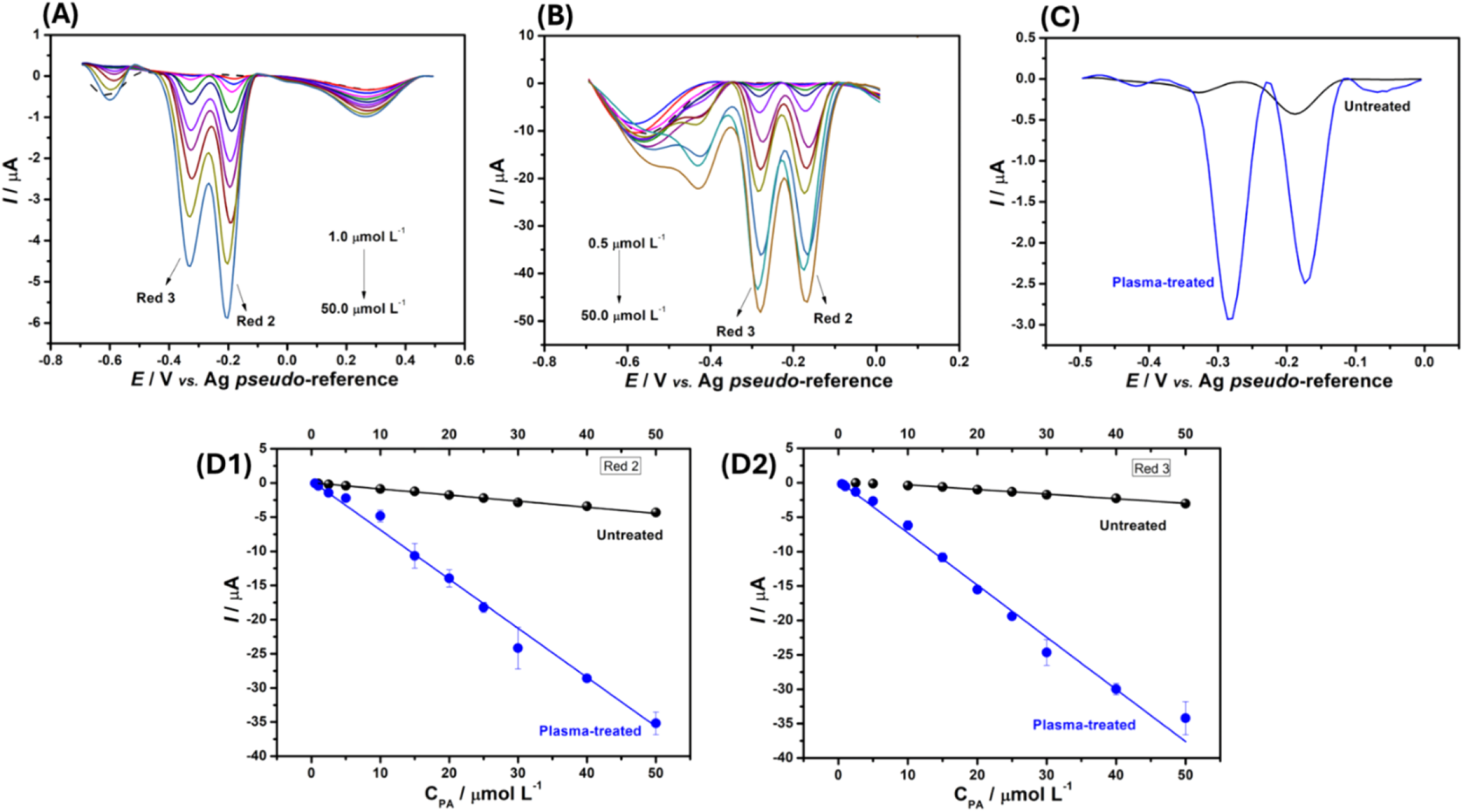
(A) Baseline-corrected SWV voltammograms (*n* = 3) performed in 0.1 mol L^–1^ HCl solution in the presence of increasing concentrations of PA (1.0–50.0 μmol L^–1^), using an untreated C-SPE. (B) Baseline-corrected SWV voltammograms (*n* = 3) carried out in the presence of increasing concentrations of PA (0.5–50.0 μmol L^–1^) in 0.1 mol L^–1^ HCl as the supporting electrolyte, employing a robotically plasma-treated C-SPE. (C) Comparison of baseline-corrected SWV responses for 5 μmol L^–1^ PA, using C-SPE before (black line) and after plasma treatment (blue line) under the same conditions. (D1, D2) Corresponding calibration plots for each electrochemical reduction process of PA Red 2 and Red 3, respectively, using both untreated (black lines) and robotic plasma-treated (blue lines) surfaces. The dashed line represents the blank signal. SWV parameters: *a*: 70 mV; Δ*E*_s_: 70 mV; *f*: 10 s^–1^.

As can be seen in Table S4, the analytical curve constructed using the plasma-treated C-SPE exhibited significantly higher sensitivity (slope of the curve) for both reduction processes studied compared to the sensitivity achieved with the untreated surface, showing a substantial increase for Red 2 (8-fold) and Red 3 (12-fold). Moreover, lower LOD values were obtained using the plasma-treated C-SPE, demonstrating improved detectability following the proposed plasma treatment, with values that were 3-fold lower for Red 2 and 22-fold lower for Red 3, compared to those achieved with the untreated surface. Indeed, when comparing the same concentration using the SWV technique (Figure 5C), the electrochemical response increased by 5-fold for Red 2 (from 0.408 to 2.227 μA) and by 20-fold for Red 3 (from 0.132 to 2.653 μA) after the treatment.

The repeatability (intraelectrode precision) of the analytical method using the plasma-treated C-SPE was evaluated through successive SWV measurements (*n* = 10) at two PA concentration levels (5 and 25 μmol L^–1^), with the results shown in Figure S16A. The relative standard deviation (RSD) values obtained for the Red 2 process were 4.1% at 5 μmol L^–1^ and 3.8% at 25 μmol L^–1^. For the Red 3 process, the RSD values were 3.3 and 3.7% at the same concentration levels, respectively, demonstrating the good precision of the proposed method. Similarly, the interelectrode precision was evaluated (Figure S16B) using three different plasma-treated C-SPEs (*n* = 3) in the presence of 25 μmol L^–1^ PA. RSD values of 4.8% for Red 2 and 4.7% for Red 3 were obtained, which are considered to be acceptable for analytical applications.

### Detection of PA in Explosive Samples Using C-SPEs

PA is an explosive compound that has been extensively used in the production of munitions and explosive charges.^49^ To demonstrate the feasibility of the proposed analytical method for PA determination and to evaluate the applicability of plasma-treated C-SPE, spike and recovery experiments were performed. Thus, PA was determined in the simulated explosive sample both before and after spiking with two concentration levels of PA, using the standard addition method (Figure S17). The procedure yielded satisfactory recovery values, ranging from 98 to 107% for the Red 2 process and from 92 to 108% for the Red 3 process of PA, confirming the reliable performance of the proposed plasma-treated C-SPE. Moreover, the results indicate that the sample matrix had no significant interference with the determination of PA. A summary of these findings is presented in Table 3.

**Table 3:** Results Obtained from the Analysis of an Explosive Sample Collected from the Granite Surface before and after Spiking with Two Known Concentrations of PA, Using Plasma-Treated C-SPE

	spiked/μmol L^–1^	measured/μmol L^–1^		(found ± SD)/μmol L^–1^		(recovery ± SD)/%	
samples	PA	Red 2 (PA)	Red 3 (PA)	Red 2 (PA)	Red 3 (PA)	Red 2 (PA)	Red 3 (PA)
A_0_	0.0	2.58 ± 0.07	2.81 ± 0.07	-	-	-	-
A_1_	2.5	5.02 ± 0.07	5.52 ± 0.08	2.44 ± 0.08	2.71 ± 0.06	98 ± 3	108 ± 1
A_2_	5.0	7.92 ± 0.10	7.72 ± 0.10	5.34 ± 0.11	4.91 ± 0.09	107 ± 2	92 ± 5

## Conclusions

This work presents an innovative robotic platform combining *X*–*Y*-axis motion control with a low-cost plasma generator, enabling fast, uniform, and reproducible surface modification of SPEs. As a proof of concept, commercial carbon SPEs were treated and analyzed using AFM, SEM, and Raman spectroscopy, showing improved morphology and structure such as increased roughness, removal of residues, and enhanced graphite crystallinity. These changes led to a higher electroactive surface area, lower charge-transfer resistance (*R*_ct_), and improved electron-transfer rate (*k* ^0^), confirming the superior electrochemical performance of the treated electrodes.

The robotic platform also enabled the reactivation of aged carbon, gold, and platinum SPEs stored for over a decade, proving its effectiveness in surface regeneration. Its rapid (∼15 s per sensor) and controlled plasma activation enable scalable production with reduced variability. The analytical performance was validated through PA detection in simulated samples, showing excellent sensitivity (up to 12-fold increase) and high accuracy (92–108%). These results highlight the platform’s potential as a scalable, efficient, and low-cost solution for SPE enhancement and regeneration.

## Supplementary Material





## Notes

Experimental procedures including chemicals and samples, electrochemical measurements, characterization methods, and details of the robotic plasma system; additional results and discussion; and supplementary figures, tables, and references (PDF)
Robotic Plasma Treatment (MP4)

## References

[1] Whitesides G. M. (2018). Soft Robotics. Angew. Chem., Int. Ed..

[2] Elpa D. P., Prabhu G. R. D., Wu S. P., Tay K. S., Urban P. L. (2020). Automation
of Mass Spectrometric Detection of Analytes and Related Workflows:
A Review. Talanta.

[3] Alden S. E., Zhang L., Wang Y., Lavrik N. V., Thorgaard S. N., Baker L. A. (2024). High-Throughput
Single-Entity Electrochemistry with
Microelectrode Arrays. Anal. Chem..

[4] Liu Z., Bian L., Shao W., Hwang S. I., Star A. (2025). An Automated
Electrolyte-Gate Field-Effect Transistor Test System for Rapid Screening
of Multiple Sensors. Digital Discovery.

[5] Burger B., Maffettone P. M., Gusev V. V., Aitchison C. M., Bai Y., Wang X., Li X., Alston B. M., Li B., Clowes R., Rankin N., Harris B., Sprick R. S., Cooper A. I. (2020). A Mobile Robotic
Chemist. Nature.

[6] Miyake, M. Electrochemical Functions. In Carbon Alloys: Novel Concepts to Develop Carbon Science and Technology; Elsevier, 2003; pp 435–445 10.1016/B978-008044163-4/50026-7.

[7] Munteanu I. G., Apetrei C. (2021). A Review on Electrochemical Sensors
and Biosensors
Used in Chlorogenic Acid Electroanalysis. Int.
J. Mol. Sci..

[8] Manikandan R., Jang H., Kim C., Yoon J., Lee J., Paik H., Chang S. (2025). Nano-Engineered
Paper-Based Electrochemical
Biosensors: Versatile Diagnostic Tools for Biomarker Detection. Coord. Chem. Rev..

[9] Azeredo N. F. B., Santos M. S. F., Sempionatto J. R., Wang J., Angnes L. (2022). Screen-Printed
Technologies Combined with Flow Analysis Techniques: Moving from Benchtop
to Everywhere. Anal. Chem..

[10] Liang G., He Z., Zhen J., Tian H., Ai L., Pan L., Gong W. (2022). Development
of the Screen-Printed Electrodes: A Mini Review on the
Application for Pesticide Detection. Environ.
Technol. Innov..

[11] Rocha D. P., Rocha R. G., Castro S. V. F., Trindade M. A. G., Munoz R. A. A., Richter E. M., Angnes L. (2022). Posttreatment of 3D-Printed
Surfaces
for Electrochemical Applications: A Critical Review on Proposed Protocols. Electrochem. Sci. Adv..

[12] Bystron T., Sramkova E., Dvorak F., Bouzek K. (2019). Glassy Carbon Electrode
Activation – A Way towards Highly Active, Reproducible and
Stable Electrode Surface. Electrochim. Acta.

[13] Chen C., Liang B., Ogino A., Wang X., Nagatsu M. (2009). Oxygen Functionalization
of Multiwall Carbon Nanotubes by Microwave-Excited Surface-Wave Plasma
Treatment. J. Phys. Chem. C.

[14] Wang S. C., Chang K. S., Yuan C. J. (2009). Enhancement
of Electrochemical Properties
of Screen-Printed Carbon Electrodes by Oxygen Plasma Treatment. Electrochim. Acta.

[15] Pankratova G., Pan J. Y., Keller S. S. (2022). Impact
of Plasma-Induced Surface
Chemistry on Electrochemical Properties of Microfabricated Pyrolytic
Carbon Electrodes. Electrochim. Acta.

[16] Pereira J. F. S., Borges P. H. S., Moura G. M., Gelamo R. V., Nossol E., Canobre S. C., Richter E. M., Munoz R. A. A. (2019). Improved Electrochemical
Performance of Pyrolytic Graphite Paper: Electrochemical versus Reactive
Cold-Plasma Activation. Electrochem. Commun..

[17] Domingo-Garcia M., Fernández-Morales I., López-Garzón F. J., Moreno-Castilla C., Pyda M. (1995). Effect of Oxygen Plasma Treatment
on the Porosity and Surface Chemical Nature of Glassy Carbons. J. Colloid Interface Sci..

[18] Pereira J. F. S., Rocha R. G., Castro S. V. F., João A. F., Borges P. H. S., Rocha D. P., de Siervo A., Richter E. M., Nossol E., Gelamo R. V., Muñoz R. A. A. (2021). Reactive
Oxygen Plasma Treatment of 3D-Printed Carbon Electrodes towards High-Performance
Electrochemical Sensors. Sens. Actuators, B.

[19] Siqueira G. P., Rocha R. G., Nascimento A. B., Richter E. M., Muñoz R. A. A. (2024). Portable
Atmospheric Air Plasma Jet Pen for the Surface Treatment of Three-Dimensionally
(3D)-Printed Electrodes. Anal. Chem..

[20] Granados-Zambrano L.
F., Korterik J. P., Estudillo-Ayala J. M., Laguna R. R., Jauregui-Vazquez D., Offerhaus H. L., Alvarez-Chavez J. A. (2024). Plasma-Based Optical Fiber Tapering
Rig. HardwareX.

[21] Wang Q., Chen A., Gao X. (2024). Sensitivity
Improvement of Laser-Induced
Breakdown Spectroscopy to Detect Heavy Metals in Water by Tesla Coil
Discharge. J. Anal. At. Spectrom..

[23] Marra M. C., Siqueira G. P., Rocha R. G., Oliveira T. C., Richter E. M., Sagás J. C., Gelamo R. V., Muñoz R. A. A. (2025). Handheld-Pen
Plasma Treatment of Graphite Sheet Electrodes for Highly Sensitive
Detection of Emerging Pollutants in Waters. J. Environ. Chem. Eng..

[24] Alba A. F., Totoricaguena-Gorriño J., Sánchez-Ilárduya M. B., Ruiz-Rubio L., Vilas-Vilela J. L., Lanceros-Méndez S., del Campo F. J. (2021). Laser-Activated
Screen-Printed Carbon Electrodes for
Enhanced Dopamine Determination in the Presence of Ascorbic and Uric
Acid. Electrochim. Acta.

[25] Yuan X., Ma L., Zhang J., Zheng Y. (2021). Simple Pre-Treatment by Low-Level
Oxygen Plasma Activates Screen-Printed Carbon Electrode: Potential
for Mass Production. Appl. Surf. Sci..

[26] Pereira J. F. S., Di-Oliveira M., Faria L. V., Borges P. H. S., Nossol E., Gelamo R. V., Richter E. M., Lopes O. F., Muñoz R. A. A. (2023). CO2-Plasma
Surface Treatment of Graphite Sheet Electrodes for Detection of Chloramphenicol,
Ciprofloxacin and Sulphanilamide. Microchimica
Acta.

[27] Pimenta M. A., Dresselhaus G., Dresselhaus M. S., Cançado L. G., Jorio A., Saito R. (2007). Studying Disorder
in Graphite-Based
Systems by Raman Spectroscopy. Phys. Chem. Chem.
Phys..

[28] Cumba L. R., Smith J. P., Brownson D. A. C., Iniesta J., Metters J. P., Do Carmo D. R., Banks C. E. (2015). Electroanalytical Detection of Pindolol:
Comparison of Unmodified and Reduced Graphene Oxide Modified Screen-Printed
Graphite Electrodes. Analyst.

[29] Randviir E. P., Brownson D. A. C., Metters J. P., Kadara R. O., Banks C. E. (2014). The Fabrication,
Characterisation and Electrochemical Investigation of Screen-Printed
Graphene Electrodes. Phys. Chem. Chem. Phys..

[30] Kwiecinska B., Suárez-Ruiz I., Paluszkiewicz C., Rodriques S. (2010). Raman Spectroscopy
of Selected Carbonaceous Samples. Int. J. Coal
Geol..

[31] Papanai G. S., Sharma I., Gupta B. K. (2020). Probing Number of
Layers and Quality
Assessment of Mechanically Exfoliated Graphene via Raman Fingerprint. Mater. Today Commun..

[32] Ghamouss F., Luais E., Thobie-Gautier C., Tessier P. Y., Boujtita M. (2009). Argon Plasma
Treatment to Enhance the Electrochemical Reactivity of Screen-Printed
Carbon Surfaces. Electrochim. Acta.

[33] Macpherson J. V. (2015). A Practical
Guide to Using Boron Doped Diamond in Electrochemical Research. Phys. Chem. Chem. Phys..

[34] Cui G., Yoo J. H., Lee J. S., Yoo J., Uhm J. H., Cha G. S., Nam H. (2001). Effect of Pre-Treatment
on the Surface
Andelectrochemical Properties of Screen-Printed Carbon Paste Electrodes. Analyst.

[35] Janus K. A., Zach M., Achtsnicht S., Drinic A., Kopp A., Keusgen M., Schöning M. J. (2025). Modification of a Bioabsorbable Carbon
Electrode on Silk-Fibroin Carriers: Setting the Composition and Adjustment
of the Working Potential. Sens. Diagn..

[36] Voiry D., Chhowalla M., Gogotsi Y., Kotov N. A., Li Y., Penner R. M., Schaak R. E., Weiss P. S. (2018). Best Practices for
Reporting Electrocatalytic Performance of Nanomaterials. ACS Nano.

[37] Lehtimäki S., Railanmaa A., Keskinen J., Kujala M., Tuukkanen S., Lupo D. (2017). Performance, Stability and Operation Voltage Optimization of Screen-Printed
Aqueous Supercapacitors. Sci. Rep..

[38] Randviir E. P. (2018). A Cross
Examination of Electron Transfer Rate Constants for Carbon Screen-Printed
Electrodes Using Electrochemical Impedance Spectroscopy and Cyclic
Voltammetry. Electrochim. Acta.

[39] Trachioti M. G., Lazanas A. C., Prodromidis M. I. (2023). Shedding
Light on the Calculation
of Electrode Electroactive Area and Heterogeneous Electron Transfer
Rate Constants at Graphite Screen-Printed Electrodes. Microchimica Acta.

[40] Lavagnini I., Antiochia R., Magno F. (2004). An Extended Method
for the Practical
Evaluation of the Standard Rate Constant from Cyclic Voltammetric
Data. Electroanalysis.

[41] Sudhakara
Prasad K., Muthuraman G., Zen J. M. (2008). The Role of Oxygen
Functionalities and Edge Plane Sites on Screen-Printed Carbon Electrodes
for Simultaneous Determination of Dopamine, Uric Acid and Ascorbic
Acid. Electrochem. Commun..

[42] Junqueira J. R. C., De Araujo W. R., Salles M. O., Paixão T. R. L.
C. (2013). Flow
Injection Analysis of Picric Acid Explosive Using a Copper Electrode
as Electrochemical Detector. Talanta.

[43] Ahmad K., Raza W., Khan R. A. (2024). Fabrication
of Picric Acid Sensor
Using Cerium Oxide-Modified Glassy Carbon Electrode. J. Mater. Sci.:Mater. Electron..

[44] Wachholz F., Biała K., Piekarz M., Flechsig G. U. (2007). Temperature Pulse
Modulated Amperometry at Compact Electrochemical Sensors. Electrochem. Commun..

[45] Castro S. V. F., Pereira J. F. S., Souza M. M. C., Siqueira G. P., Santana M. H. P., Richter E. M., Munoz R. A. A. (2024). Rapid
Sequential
Determination of the Explosives 2,4,6-Trinitrotoluene and Cyclotrimethylenetrinitramine
in Forensic Samples Employing a Graphite Sheet Sensor and Cyclic Square-Wave
Stripping Voltammetry. Microchimica Acta.

[46] Bratin K., Kissinger P. T., Briner R. C., Bruntlett C. S. (1981). Determination
of Nitro Aromatic, Nitramine, and Nitrate Ester Explosive Compounds
in Explosive Mixtures and Gunshot Residue by Liquid Chromatography
and Reductive Electrochemical Detection. Anal.
Chim. Acta.

[47] Zhang H. X., Cao A. M., Hu J. S., Wan L. J., Lee S. T. (2006). Electrochemical
Sensor for Detecting Ultratrace Nitroaromatic Compounds Using Mesoporous
SiO2-Modified Electrode. Anal. Chem..

[48] Mocak J., Bond A. M., Mitchell S., Scollary G. (1997). A Statistical Overview
of Standard (IUPAC and ACS) and New Procedures for Determining the
Limits of Detection and Quantification: Application to Voltammetric
and Stripping Techniques. Pure Appl. Chem..

[49] Fabin M., Łapkowski M., Jarosz T. (2023). Methods for Detecting Picric Acid—A
Review of Recent Progress. Appl. Sci..

