# Supplementary material for: Robotic Plasma System for Rapid Activation of Mass-Produced Electrochemical Sensors
Source: Anal Chem. 2025 Dec 1;98(3):1915–27. doi: 10.1021/acs.analchem.5c04556 (PMC12856828; doi:10.1021/acs.analchem.5c04556)
Supplement: Supplementary file 1 [file ac5c04556_si_001.pdf]

SUPPORTING INFORMATION FOR:

Robotic Plasma System for Rapid Activation of Mass-Produced Electrochemical Sensors

Marina Di-Oliveira, Mariana C. Marra, Raquel G. Rocha, Teodoro R. Terra, Rodrigo A. A. Muñoz*, Eduardo M. Richter*

Institute of Chemistry, Federal University of Uberlândia, 38408-100, Uberlândia, Brazil

Supporting Information

S1. Experimental section……………………………………………………………………S2
S1.1 Chemicals and samples ……………………………………………………………………S2
S1.2 Electrochemical measurements…………………………………………………………...S3
S1.3 Characterization……………………………………………………………………………S4
S1.4 Robotic plasma system……………………………………………………………………...S5
S2. Results and discussion section …………………………………………………………......S9
S3. References…………………………………………………………………………………...S22

***Corresponding author:**

munoz@ufu.br

emrichter@ufu.br

S1. Experimental section

S1.1 Chemicals and samples

All aqueous solutions were prepared using deionized water with resistivity not less than 18 MΩ cm (Millipore Direct-Q3 water purification, MA, USA). Potassium ferricyanide (99% w/w), sodium nitrite (98% w/w), sodium chloride (99% w/w), and paracetamol (98% w/w) were acquired from Labsynth (Diadema, Brazil). Uric acid (≥99% w/w) and dopamine hydrochloride (98% w/w) were obtained from Sigma Aldrich (St Louis, USA). Potassium chloride (≥99.5% w/w) and tyrosine (99% w/w) were purchased from Êxodo Cientifica (São Paulo, Brazil). Sodium phosphate dibasic (99% w/w), citric acid (≥98% w/w), sodium sulfate (99% w/w), potassium thiocyanate (99% w/w), ascorbic acid (99% w/w), acetic acid (99% w/v) and phosphoric acid (85% w/v) were obtained from Vetec (Rio de Janeiro, Brazil). Boric acid (99% w/w) was obtained from AppliChem Panreac (Barcelona, Spain). Sodium hydroxide (98% w/w) from ChemiFlex (São Bernardo do Campo, Brazil). All reagents were analytical grade and employed without further purification.

A mixture of phosphoric, acetic, and boric acids (both at 0.04 mol L^{-1}) was employed for preparing Britton-Robinson (BR) buffer solutions. The pH adjustment of BR solutions was performed using 1 mol L^{-1} NaOH solution, prior to use as a supporting electrolyte.

An amount of 1 mg of explosive picric acid (PA)was weighed and spread on a granite laboratory bench. The residue was then collected using a swab and quantitatively transferred to a vial containing 1 mL of acetonitrile. An aliquot of this solution was subsequently diluted in supporting electrolyte prior to analysis. The method’s accuracy was evaluated through addition-recovery studies. For this purpose, the simulated explosive samples were spiked with different concentrations of PA, and the PA content was determined using the standard addition method.

S1.2 Electrochemical measurements

All electrochemical measurements, including cyclic voltammograms (CV), square-wave voltammetry (SWV), and electrochemical impedance spectroscopy (EIS), were carried out using a PGSTAT204N or PGSTAT101 potentiostat/galvanostat (Metrohm Autolab BV, Utrecht, The Netherlands). Data acquisition and processing (baseline correction for SWV scans) were performed using NOVA 2.1.7 software. Electrochemical experiments were accomplished using commercial SPEs (DRP-110, Metrohm DropSens, Oviedo, Spain), which include a three-electrode system printed on the same strip. The strips displayed a 4 mm diameter disk of a carbon screen-printed as a working electrode, a carbon counter electrode, and a silver pseudo-reference electrode. Additionally, screen-printed electrodes (SPEs) composed of different materials and stored for over a decade were employed to investigate the impact of plasma treatment. The tested materials included gold (254AT and 220BT which are produced by curing at high and low temperature, respectively, according to fabricant), platinum (550), and dual working carbon (C1110) electrode, all sourced from Metrohm DropSens (Oviedo, Spain).

Furthermore, a 3D-printed working electrode was fabricated following methodologies previously described by our research group.[1] It was employed to investigate whether robotic plasma treatment could enhance the electrochemical activity of other types of electrodes that can be produced on a large scale and may suffer from inter-electrode reproducibility issues or require a surface activation step. Interestingly, 3D-printed electrodes, particularly those produced using commercial conductive filaments, meet all these criteria. Accordingly, a commercial filament of carbon black and polylactic acid (CB/PLA) (Protopasta®, WA, USA) was used to produce the 3D-printed CB/PLA electrodes. The printing process was carried out using a fused deposition modeling (FDM) 3D printer (Flashforge Dreamer NX, China) under the following parameters: layer height of 0.05 mm, nozzle diameter of 0.6 mm, printing speed of 70 mm s^{-1} in a horizontal orientation, bed temperature of 90 °C, and extruder temperature of 220 °C. The geometry of the device corresponds to that reported previously, consisting of a circular working electrode connected to an extension designed for electrical contact.

Figure S11 shows images of the 3D printed CB/PLA working electrode before and after treatment with the robotic plasma system.

EIS measurements were performed at the half-wave potential ($E_{1/2}$) of the redox probe (+0.23 V *vs.* Ag|AgCl|$KCl_{(sat.)}$ for the untreated surface and +0.18 V *vs.* Ag|AgCl|$KCl_{(sat.)}$ for the plasma-treated surface) in the presence of 1 mmol L^{-1} $[Fe(CN)_6]^{3-/4-}$ in 0.1 mol L^{-1} KCl solution, applying an alternating potential with an amplitude value of 10 mV in a frequency range from 50 kHz to 0.1 Hz. The equivalent Randles circuit was used to fit the experimental results and to determine the charge transfer resistance (Rct) related to the $[Fe(CN)_6]^{3-/4-}$ species.

The double-layer capacitance (Cdl) was calculated by CV measurements ranging from 0.00 to +0.30 V (*vs.* Ag *pseudo*-reference) at different scan rates in 0.1 mol L^{-1} KCl solution according to protocols from the literature.[2] A plot of the difference between the anodic and cathodic currents at +0.15 V normalized by geometric area (A_{geo} = 0.11 cm^2) and different scan rate (5.0- 30.0 mV s^{-1}). The Cdl is the slope of the linear curve. The electrochemically active surface area (A_{ele}) is directly proportional to the Cdl, as shown in the following equation:

$$A_{ele} = \frac{C_{dl}\ A_{geo}}{Cs} \quad \text{(S1)}$$

where Cs is the specific capacitance and A_{geo} is the geometric area.

The electron transfer kinetics at the electrode surface of both untreated and plasma-treated C-SPEs was assessed by cyclic voltammetry in the presence of 1.0 mmol L^{-1} $[Ru(NH_3)_6]^{2+/3+}$ in 0.1 mol L^{-1} KCl solution, while varying the scan rate (v) from 10 to 200 mV s^{-1}. This approach enables the estimation of the k^0, since the ΔEp values obtained at different v values can be used to determine the corresponding Ψ values for each condition. Subsequently, k^0 can be graphically determined by linear regression, as described by Lavagnini *et al.*[3]

$$\Psi = (-0.6288+0.0021\Delta Ep)/(1-0.017\Delta Ep) \quad \text{(S2)}$$

$$\Psi = k^0[\pi \mathrm{DnF}v/\mathrm{RT}]^{-\frac{1}{2}}$$

(S3)

Where D is the diffusion coefficient of $[Ru(NH_3)_6]^{2+/3+}$, n is the number of electrons transferred in the electrochemical reaction, R = 8.314 J mol^{-1} K^{-1}, T = 298 K, and F (Faraday's constant) = 96,485 C mol^{-1}.

S1.3 Characterization

Raman spectra were obtained using a HORIBA spectrometer, model LabRAM HR evolution, with OSD Syncerity detector, with laser (λ = 532 nm). SEM images were collected using a microscope Tescan VEJA 3 LMU, operating at 20 kV. Atomic Force microscopy (AFM) images were obtained using a scanning probe microscope (SPM-9600, Shimadzu, Japan), in dynamic force mode and employing silicon probes PPP-NCHR AFM (Nanosensors™, Switzerland) with resonance frequency of 330 kHz, force constant of 42 N m^{-1}, length 125 µm, mean width 30 µm and thickness 4 µm.

Surface water contact angle measurements of each electrode were performed using a smartphone camera, mounted on a universal holder to ensure a stable and reproducible imaging position. Both untreated and plasma-treated SPEs were held within the camera's focal plane in a fixed setup using a cable connector (BICAST model, Metrohm DropSens, Oviedo, Spain) on a leveled horizontal surface. A droplet of deionized water (50 µL) was deposited on the working electrode, and images were captured 10 s after deposition. The contact angle of each droplet was measured on both the left and right sides using GeoGebra® software (https://www.geogebra.org/classic), and the average value was subsequently calculated.[4]

S1.4 Robotic plasma system

Adjustments were made to the communication between the Raspberry Pi and the SKR MINI E3 V2.0 controller board by establishing a serial port connection. This configuration enabled the software running on the Raspberry Pi to transmit movement and plasma activation commands directly to the controller. The communication speed was set to 115200 baud, ensuring adequate response time for real-time control. As the system does not utilize extruders or temperature sensors, the firmware was modified to disable the outputs associated with these components, avoiding potential conflicts. Similarly, other unused peripherals were deactivated to improve system efficiency. To minimize electromagnetic interference from the power network and platform components, 5 mm ferrite filters were applied to the connections of all critical elements, including the interface, power supplies, and plasma generator.

The developed system provides a precise and highly efficient approach for the surface treatment of SPEs, with the potential to enhance both electrochemical performance and surface activation. By integrating robotic motion along the X and Y axes, the platform ensures uniform plasma exposure, effectively eliminating inconsistencies and operational errors commonly associated with manual treatment methods. Constructed using readily available and cost-effective components, the entire setup was assembled at a total cost of approximately USD $412—substantially lower than that of commercially available plasma treatment systems. This makes the proposed solution not only technically robust and reproducible, but also economically viable and scalable for broader laboratory and industrial applications. Photographs of each component of the robotic system are presented in **Figure S1**, and a detailed breakdown of material costs is provided in **Table S1**.

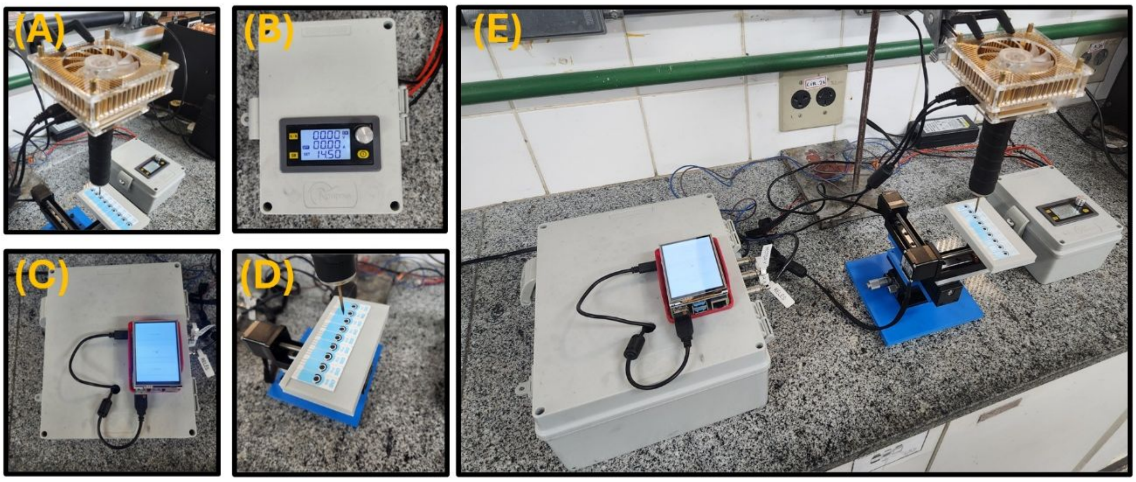

Figure S1. Photographs of the components comprising the proposed robotic plasma treatment system: **(A)** Music Tesla coil arc plasma source; **(B)** Power control unit for plasma generation; **(C)** Graphical user interface for operational control; **(D)** Treatment platform with semi-automated X–Y axis motion; **(E)** Fully assembled robotic plasma treatment system.

Table S1. List of components and their respective costs for assembling the robotic plasma treatment system.

**Components**	***Cost (USD)**
DC8–32V Music Tesla	49
48V 5A power supply	20
XY6020L power supply regulator	30
NEMA 11 CNC (y axis)	35
NEMA 11 CNC (x axis)	44
Micrometer-adjustment platform	38
BIGTREETECH SKR MINI E3 V2.0	36
Raspberry Pi 4 Model B	127
3.5-inch Touch Screen LCD Display	20
Ferrite filters	3
**Total of cost**	412

*These values are based on Amazon.com.

To enable the use of an educational plasma generation kit for efficient activation of SPE surfaces, the power supply section of the electrical circuit was modified (**Figure S2A**). This modification involved removing a circuit component originally intended for audio input, which enabled voice-modulated plasma generation (**dashed line – Figure S2B**). Its removal enhanced activation of the power transistor, thereby optimizing both the output power and the stability of the plasma discharge (**Figure S2C**). As a result, the device offered improved control over the plasma, making it more stable and better suited for electrode surface treatment.

The board's firmware was reconfigured to repurpose one of the hot end heater outputs, typically used for temperature control in 3D printers, as a switching signal for the plasma generator. This adaptation enabled a reliable and programmable interface for precise control of plasma discharge during the electrode treatment process. This output was reconfigured within the Marlin firmware (for the SKR MINI E3 V2.0 board) to function as a digital control pin capable of delivering real-time actuation signals. Marlin is an open-source firmware originally developed for controlling 3D printers based on Arduino-compatible microcontrollers. It interprets G-code commands and manages precise motion control, temperature regulation, and peripheral devices such as fans and heaters. Although originally tailored for 3D printing, Marlin is highly customizable and can be adapted for a variety of applications involving precise motor control and digital signal management. In this implementation, the firmware was extensively modified to remove 3D printer-specific functions and integrate routines for X and Y axis control as well as plasma actuation. Plasma generation was programmed to respond to the M106 and M107 G-code commands—originally intended for fan control—where M106 activates and M107 deactivates the plasma. This customization enabled efficient control of the plasma system while retaining Marlin's core motion control capabilities, providing a simple yet effective solution for precise and synchronized plasma discharge during robotic surface treatment.

Additionally, Raspberry Pi 4 Model B is used to manage the graphical interface and generate G-code commands, which are then transmitted to the controller board. The system includes a 3.5-inch touchscreen LCD display for the Raspberry Pi 4, enabling

direct user interaction. This interface allows intuitive configuration of system parameters, such as defining the work area and adjusting treatment settings. The Raspberry Pi acts as a bridge between the user and the control hardware, providing a seamless means to visualize and modify treatment conditions in real time.

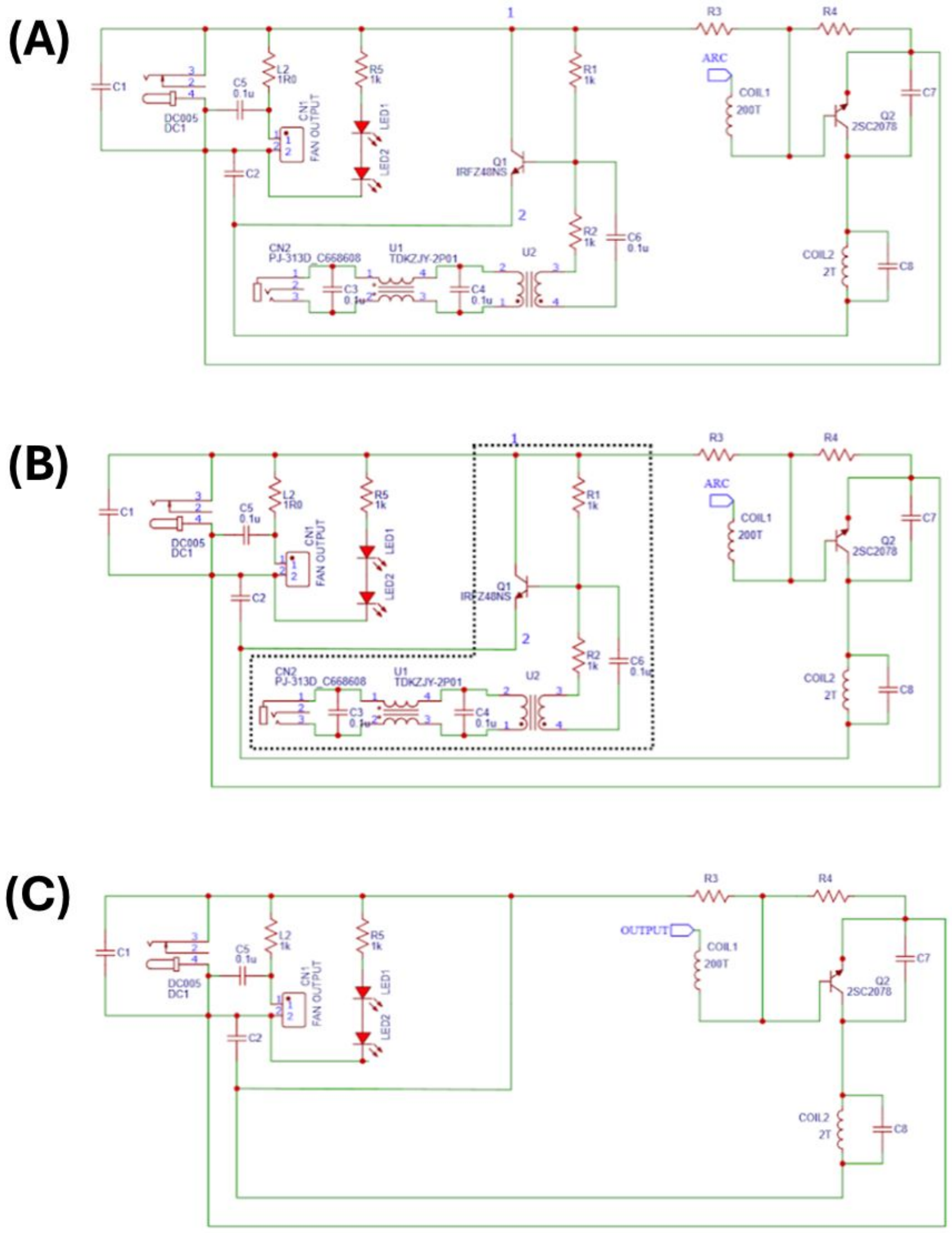

Figure S2. (A) Original electrical schematic diagram of the plasma generator, in which an audio input modulated the plasma discharge. **(B)** The dashed section indicates the portion removed to adapt the power supply for integration into the developed system; this

modification involved eliminating the highlighted circuit and directly connecting point 1 to point 2. **(C)** Final operating circuit used in the robotic plasma treatment system.

S2. Results and discussion section

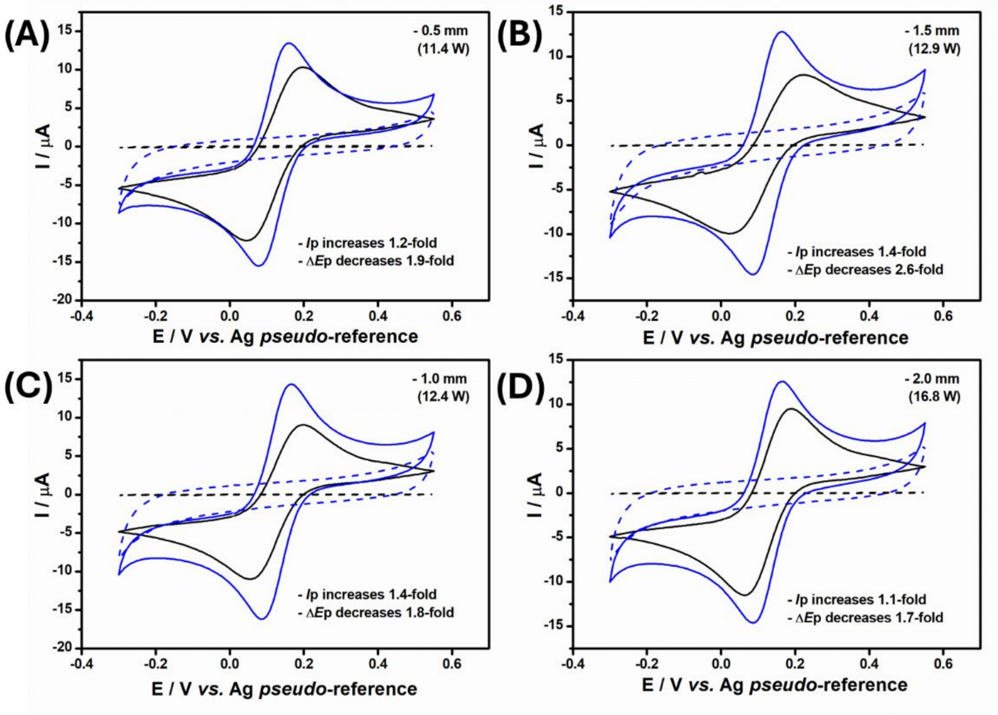

Figure S3. Effect of the distance between the lab-built robotic plasma system (Tesla coil tip) and the electrode surface on the cyclic voltammetric response of 1.0 mmol L^{-1} $[Fe(CN)_6]^{3-/4-}$ in 0.1 mol L^{-1} KCl, using C-SPEs before (black lines) and after (blue lines) plasma treatment. Distances evaluated: **(A)** 0.5 mm, **(B)** 1.0 mm, **(C)** 1.5 mm, and **(D)** 2.0 mm. Dashed lines represent blank signals. Scan rate = 50 mV s^{-1}; step potential = 5 mV.

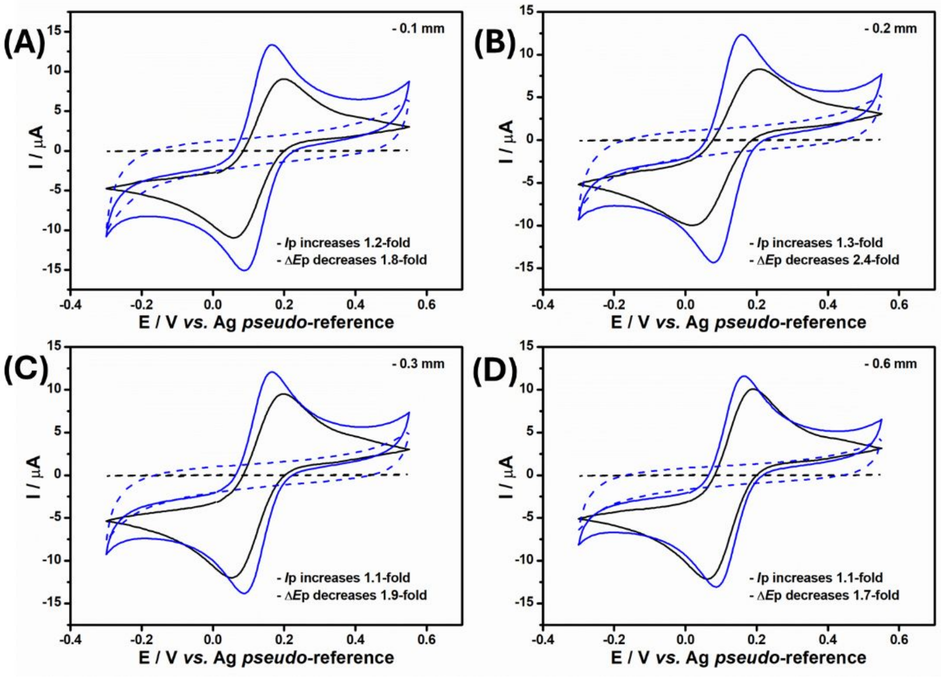

Figure S4. Effect of the spacing between plasma lines, reproducibly applied by the lab-built robotic plasma system, on the cyclic voltammetric response of 1.0 mmol L^{-1} $[Fe(CN)_6]^{3-/4-}$ in 0.1 mol L^{-1} KCl, using C-SPEs before (black lines) and after (blue lines) plasma treatment. Line spacings: **(A)** 0.1 mm, **(B)** 0.2 mm, **(C)** 0.3 mm, and **(D)** 0.6 mm. Dashed lines represent blank signals. Scan rate = 50 mV s^{-1}; step potential = 5 mV.

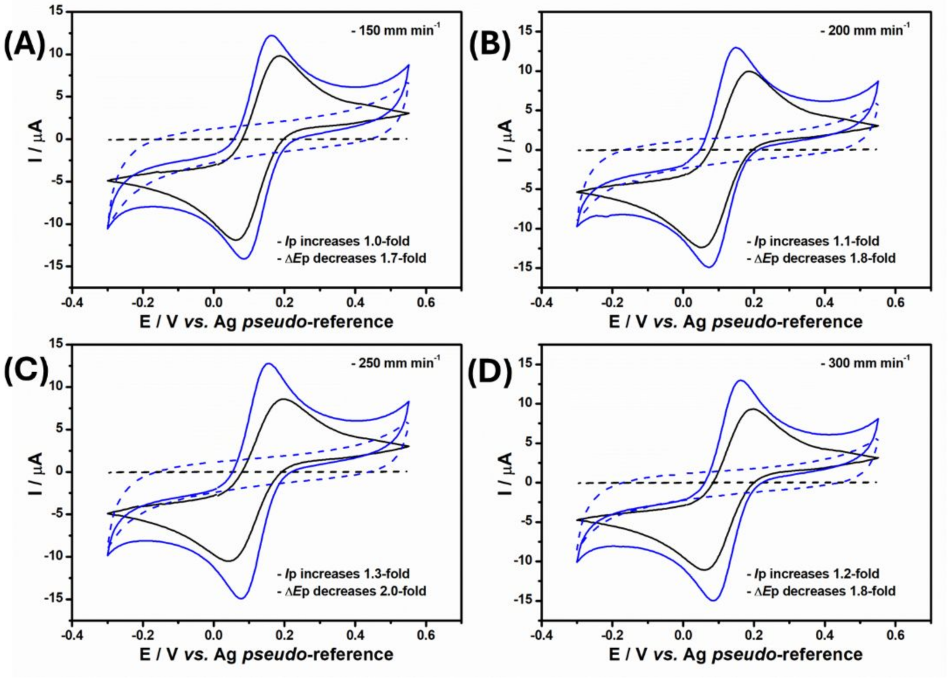

Figure S5. Effect of the treatment rate of the lab-built robotic plasma system on the cyclic voltammetric response of 1.0 mmol L^{-1} $[Fe(CN)_6]^{3-/4-}$ in 0.1 mol L^{-1} KCl, using C-SPEs before (black lines) and after (blue lines) plasma treatment. Treatment speeds: **(A)** 150 mm·min^{-1}, **(B)** 200 mm·min^{-1}, **(C)** 250 mm·min^{-1}, and **(D)** 300 mm·min^{-1}. Dashed lines represent blank signals. Scan rate = 50 mV·s^{-1}; step potential = 5 mV.

Table S2. Summary of the effects of treatment parameters on the cyclic voltammetric response (Epa and ΔEp values) of 1.0 mmol L^{-1} $[Fe(CN)_6]^{3-/4-}$ in 0.1 mol L^{-1} KCl.

Treatment parameters	Value	Untreated SPE (control)		Plasma-treated SPE	
		Δ*E*p / mV	*I*pa / µA	Δ*E*p / mV	*I*pa / µA
Treatment distance / mm	**0.5**	135.96 ± 5.00	11.10 ± 0.07	70.50 ± 0.01	13.60 ± 0.06
	**1.0**	130.92 ± 5.00	9.80 ± 0.39	70.49 ± 0.03	13.90 ± 0.12
	**1.5**	174.56 ± 2.90	8.38 ± 0.30	67.14 ± 2.91	12.00 ± 0.23
	**2.0**	115.81 ± 0.01	10.70 ± 0.17	67.13 ± 2.80	12.00 ± 0.27
Distance between treatment lines / mm	**0.1**	129.00 ± 2.90	9.84 ± 0.27	70.50± 0.03	12.20 ± 0.48
	**0.2**	166.00 ± 0.02	8.89 ± 0.19	68.88 ± 2.91	11.70 ± 0.57
	**0.3**	132.60 ± 3.00	10.50 ± 0.29	68.82 ± 2.92	11.40 ± 0.51
	**0.6**	117.50 ± 2.92	10.08 ± 0.18	68.82 ± 2.91	11.29 ± 0.15
Treatment rate / mm min^{-1}	**150**	114.14 ± 2.91	10.90 ± 0.12	65.46 ± 0.01	11.00 ± 0.79
	**200**	124.00 ± 2.90	11.10 ± 0.04	68.82 ± 2.91	12.10 ± 0.50
	**250**	141.00 ± 5.00	9.38 ± 0.13	68.82 ± 2.90	11.80 ± 0.81
	**300**	124.00 ± 5.81	10.20 ± 0.08	68.82 ± 2.91	12.20 ± 0.66

Since the plasma generator operates by exciting atmospheric gases through an electric field applied between a cathode and an anode,[1] this distance directly affects the power delivered by system. Consequently, the distance between the tip of the plasma source and the electrode surface was the first parameter evaluated. As observed in **Figure S3** and **Table S2**, all plasma-treated electrodes presented improved values of Ipa and ΔEp in comparison with control experiments. Analyzing each selected distance of plasma treatment, slightly higher current values for shorter distance treatments (0.5 or 1.0 mm) while slightly lower ΔEp were verified for larger distances (1.5 or 2.0 mm). Therefore, any value could be selected for further experiments. We selected a 1.5 mm distance between the plasma tip and the electrode surface, at an applied power of 12.9 W.

Next, the effect of the plasma activation protocol by varying the spacing between adjacent scan lines over the electrode surface was evaluated. As shown in **Figure S4 and Table S2**, once again, no significant variation of Ipa and ΔEp was noted for the plasma-treated SPEs. Similarly, when the rate of plasma treatment was varied from 150 to 300 mm min^{-1}, no significant changes of Ipa and ΔEp was verified. Therefore, we can affirm that the robotic plasma system is robust considering that small variations of the operating parameters did not cause substantial changes of the voltametric profile of the redox probe, consequently, did not variate Ipa and ΔEp values. Moreover, it is clear in **Figures S3, S4, S5** and from the data in **Table S2** that the plasma treatment provided improved electrochemical activity of the SPEs. The commercial SPEs without any surface treatment presented typical voltametric behavior for $[Fe(CN)_6]^{3-/4}$, however, variation of Ipa and ΔEp values were verified (important to mention that different brand-new electrodes were used in this experiment). Commercially available SPEs are often coated with binders, and other contaminants originating from the conductive ink-based production process. Moreover, their surfaces are inherently non-uniform between devices, even within the same batch, due to variations in binder concentration, ink layer thickness, and distribution of the conductive material.[5] Consequently, bare SPEs exhibit intrinsic reproducibility issues, highlighting the need for surface treatment prior to use.

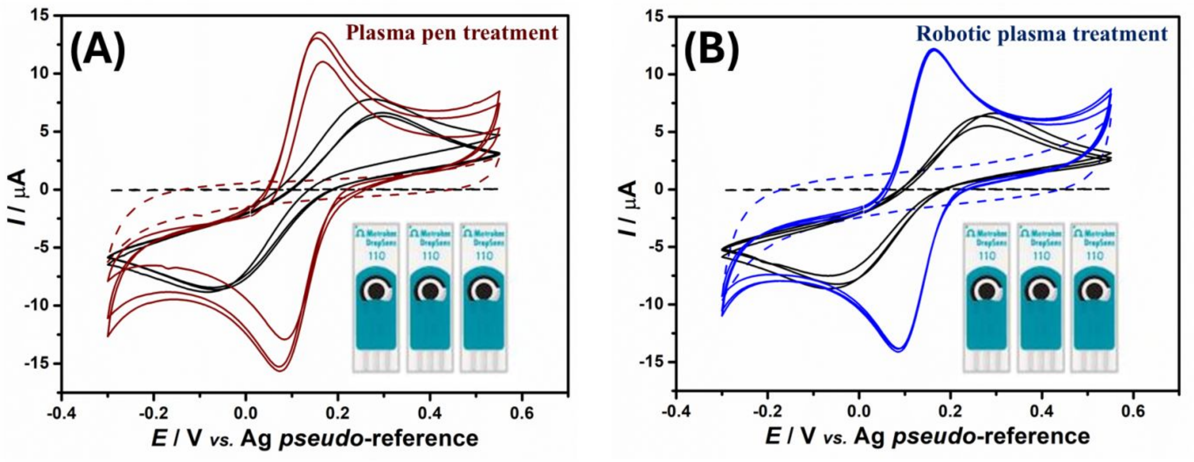

Figure S6. **(A)** CV responses before (black lines) and after plasma-treatment (wine lines) for different C-SPEs (n = 3), in the presence of the 1.0 mmol L^{-1} $[Fe(CN)_6]^{3-/4-}$ in 0.1 mol L^{-1} KCl solution. Two voltammograms (before and after plasma treatment) were obtained for each C-SPE, with the surface activation performed manually by a different analyst from our research group for each electrode, employing the plasma pen treatment conditions previously optimized and reported by Siqueira *et al.*[1] **(B)** CV responses obtained before (black lines) and after robotic plasma treatment (blue lines) for C-SPEs under the same experimental conditions, using three different electrodes from the same batch as in **(A)**. The dashed lines correspond to the blank signals obtained from an untreated and a plasma-treated SPE. Scan rate=50 mV s^{-1}; step potential = 5 mV.

Table S3. ΔEp and *I*pa values for plasma-pen-treated C-SPEs (n = 3). Additionally, the table presents the mean values of each parameter, along with the corresponding RSDs, calculated from the three electrodes. These values were obtained from CV responses recorded in the presence of 1.0 mmol L^{-1} $[Fe(CN)_6]^{3-/4-}$ in a 0.1 mol L^{-1} KCl solution.

Air plasma-pen treated C-SPE		
**C-110**	**ΔEp / mV**	**Ipa / µA**
**1**	83.9	13.6
**2**	78.6	13.0
**3**	81.8	11.1
**Mean ± SD**	81.4 ± 2.7	12.6 ± 1.3
**RSD / %**	3.3	10.4

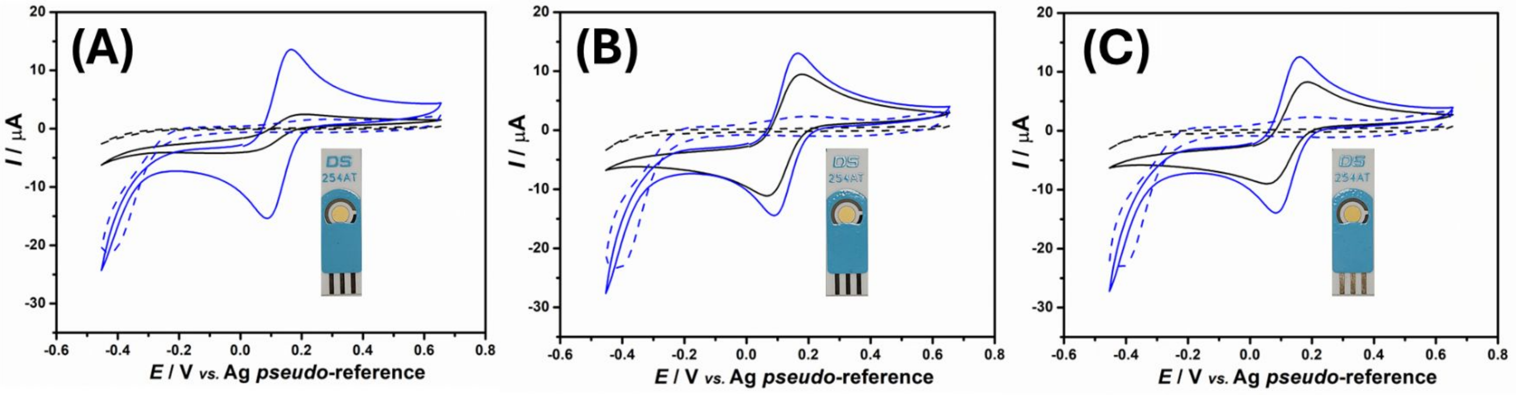

Figure S7. CV responses recorded in the presence of 1.0 mmol L^{-1} $[Fe(CN)_6]^{3-/4-}$ in 0.1 mol L^{-1} KCl solution, for three aged gold AT 254AT-SPEs (**A, B, and C**) that had been stored for more than 10 years, before (black lines) and after (blue lines) plasma treatment. The dashed lines correspond to the respective blank signals. Scan rate = 50 mV s^{-1}; step potential = 5 mV.

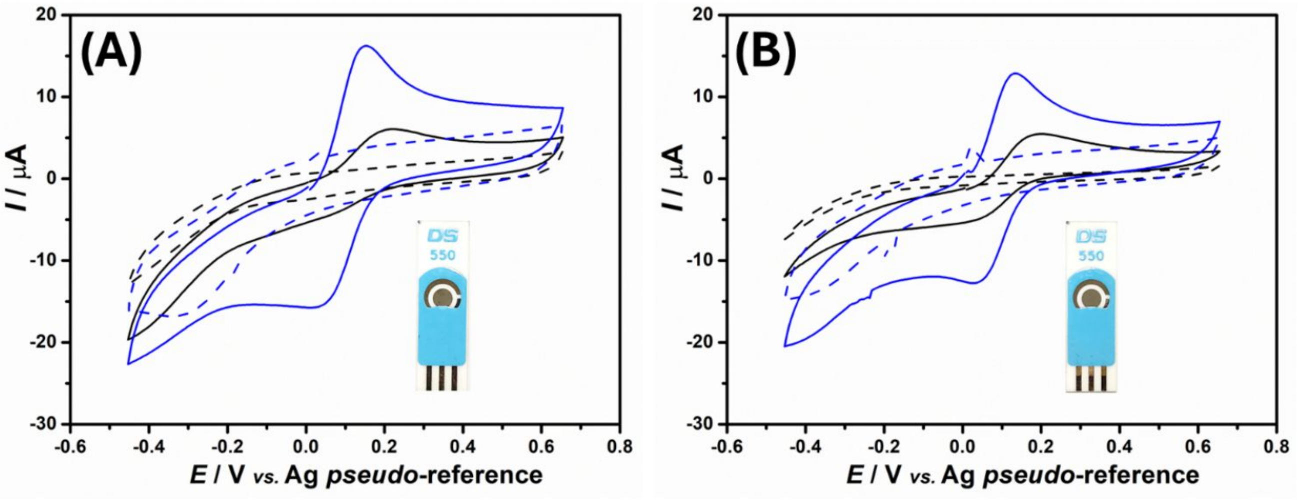

Figure S8. CV responses recorded in the presence of 1.0 mmol L^{-1} $[Fe(CN)_6]^{3-/4-}$ in 0.1 mol L^{-1} KCl solution, for two aged platinum-SPEs (550) (**A and B**) that had been stored for more than 10 years, before (black lines) and after (blue lines) plasma treatment. The dashed lines correspond to the respective blank signals. Scan rate=50 mV s^{-1}; step potential = 5 mV.

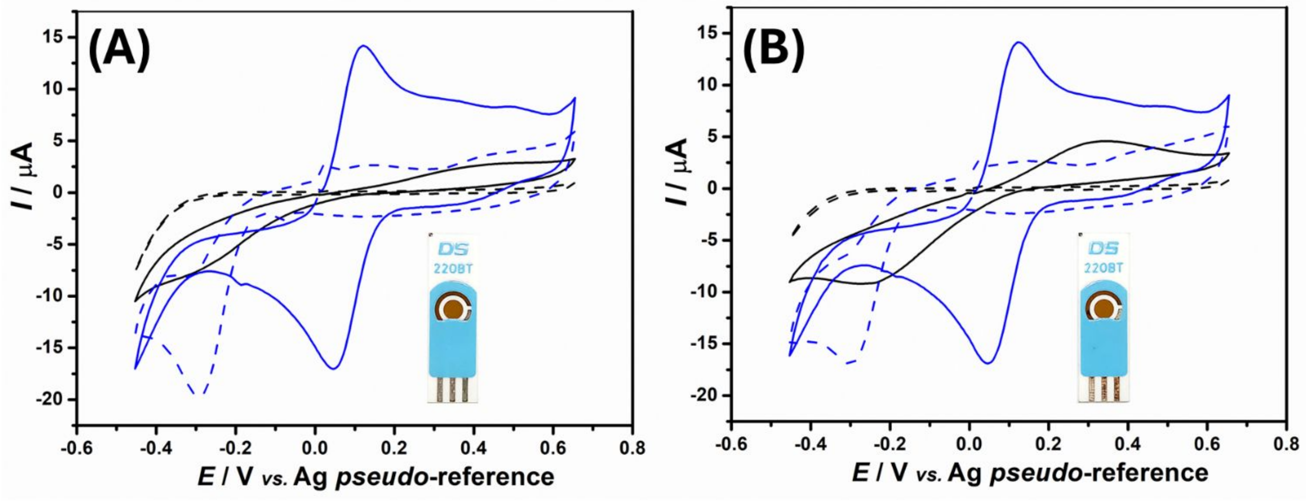

Figure S9. CV responses recorded in the presence of 1.0 mmol L^{-1} $[Fe(CN)_6]^{3-/4-}$ in 0.1 mol L^{-1} KCl solution, for two aged gold 220BT-SPEs (**A and B**) that had been stored for more than 10 years, before (black lines) and after (blue lines) plasma treatment. The dashed lines correspond to the blank signals. **S**can rate=50 mV s^{-1}; step potential = 5 mV.

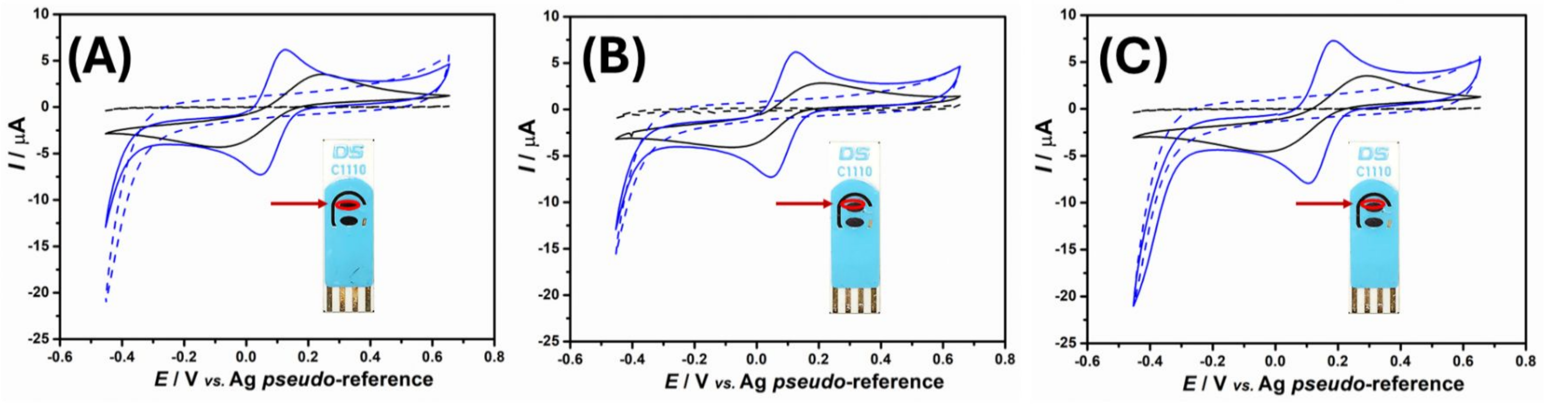

Figure S10. CV responses recorded in the presence of 1.0 mmol L^{-1} $[Fe(CN)_6]^{3-/4-}$ in 0.1 mol L^{-1} KCl solution, for three carbon aged C1110-SPEs (**A, B, and C**) that had been stored for more than 10 years, before (black lines) and after (blue lines) plasma treatment. Measurements and plasma treatment were conducted using only the top working electrode (WE). Scan rate=50 mV s^{-1}; step potential = 5 mV.

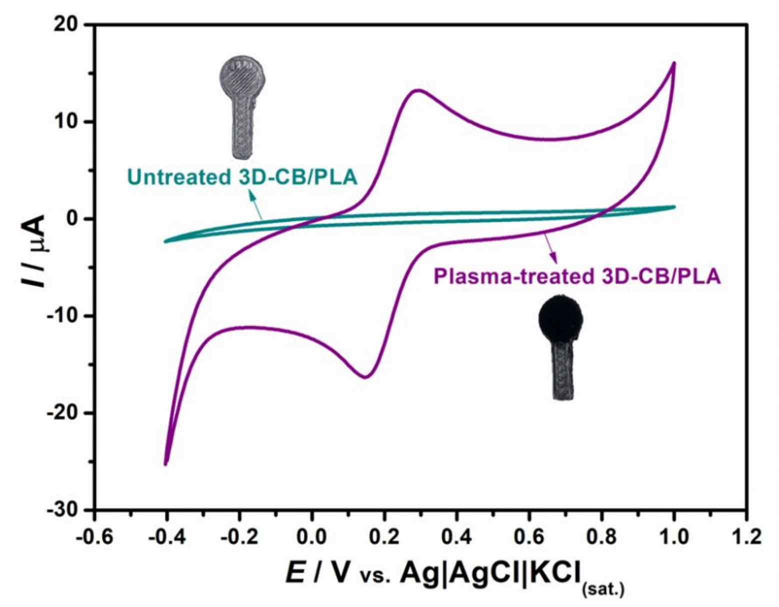

Figure S11. Effect of the lab-built robotic plasma system treatment on the cyclic voltammetric response of 1.0 mmol L^{-1} $[Fe(CN)_6]^{3-/4-}$ in 0.1 mol L^{-1} KCl, using a 3D-printed CB/PLA electrode before (green line) and after (purple line) plasma treatment. The plasma activation was performed under the same optimized conditions established for the SPEs in this work. CV conditions: scan rate = 50 mV s^{-1}; step potential = 5 mV.

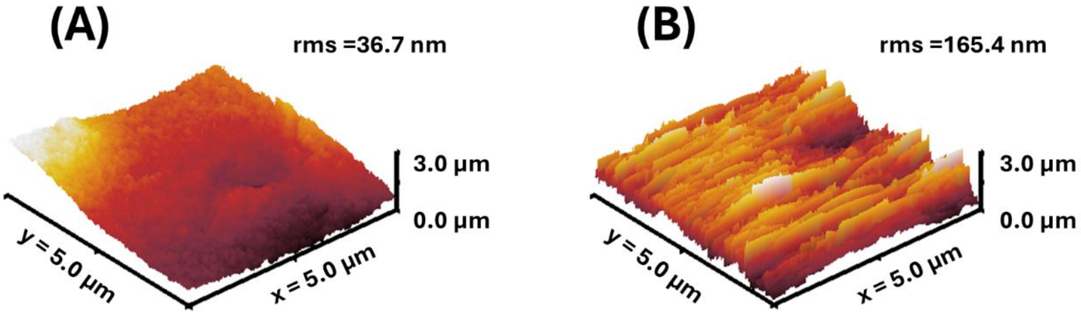

Figure S12. AFM images of **(A)** untreated and **(B)** robotic air plasma-treated C-SPE.

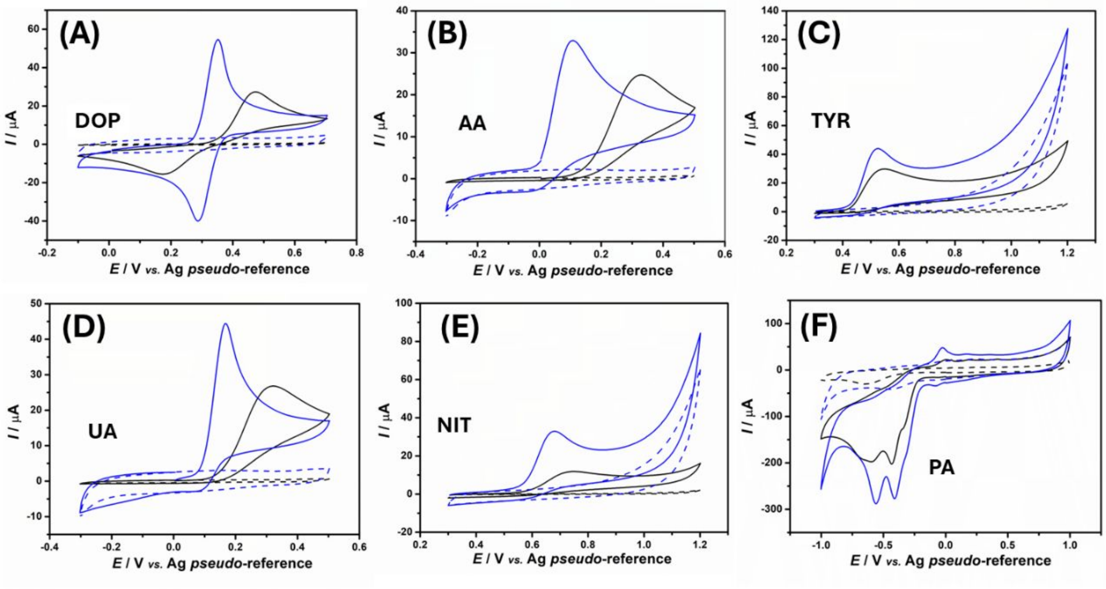

Figure S13. Cyclic voltammograms recorded using C-SPEs before (black lines) and after robotic plasma treatment (blue lines) in the presence of: **(A)** 1.0 mmol L^{-1} dopamine (DOP) in 0.1 mol L^{-1} $HClO_4$, **(B)** 1.0 mmol L^{-1} ascorbic acid (AA) in 0.1 mol L^{-1} acetate buffer solution (pH 4.0), **(C)** 1.0 mmol L^{-1} tyrosine (TYR) in BR buffer solution (pH 7.0), **(D)** 1.0 mmol L^{-1} uric acid (UA) in 0.1 mol L^{-1} acetate buffer solution (pH 4.0), **(E)** 1.0 mmol L^{-1} nitrite (NIT) in BR buffer solution (pH = 2), and **(F)** 1.0 mmol L^{-1} picric acid (PA) in 0.1 mol L^{-1} phosphate buffer solution (pH 2.0). CV conditions: Scan rate = 50 mV s^{-1}; step potential = 5 mV.

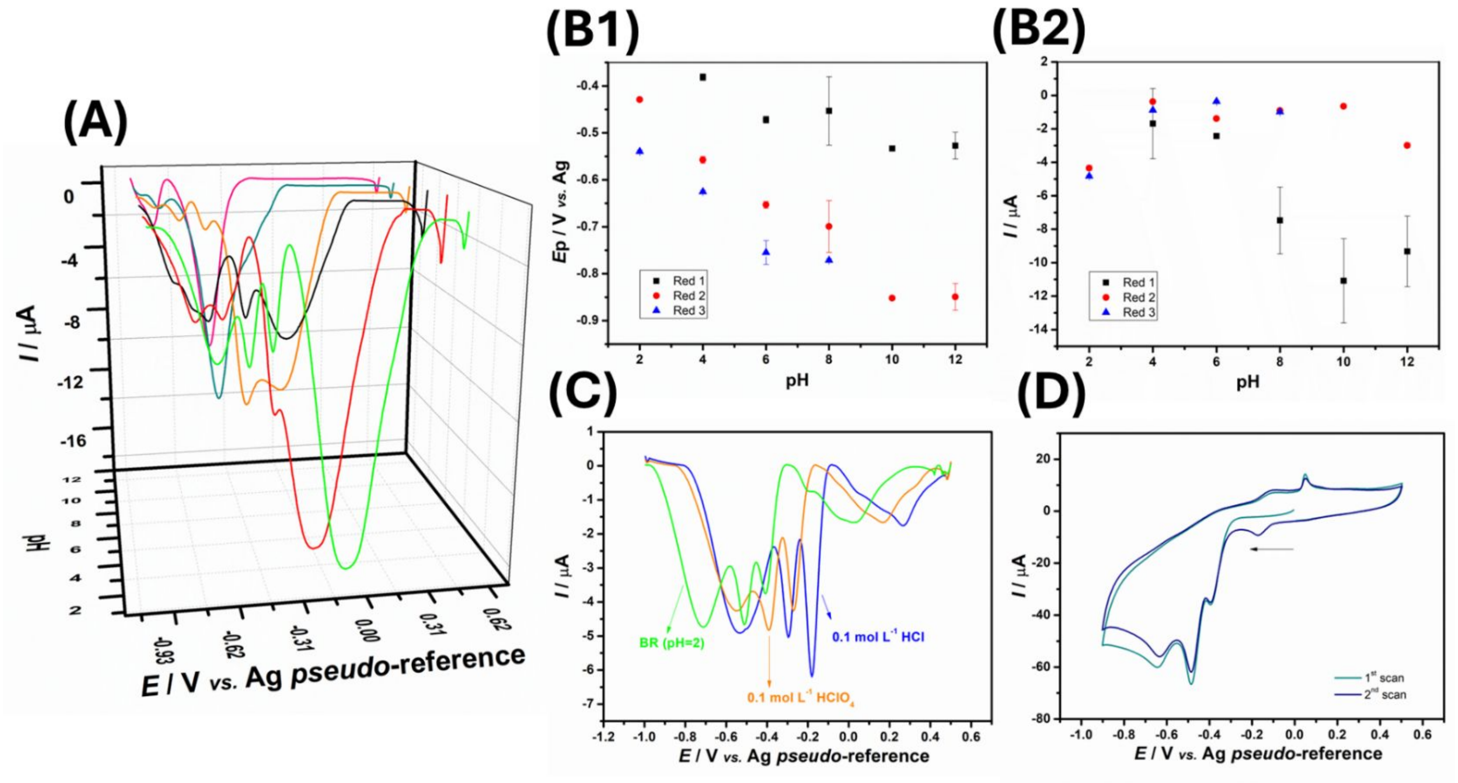

Figure S14. (A) Baseline-corrected SWV responses showing the effect of pH (2.0 – 12.0) on the electrochemical response for 30 µmol L^{-1} PA, using BR buffer solution. **(B1)** and **(B2)** are plots of peak potential (*E*p) and peak current (*I*p) *versus* pH obtained for PA, respectively. **(C)** Baseline-corrected SWV recorded on the plasma-treated C-SPE in the presence of 30 µmol L^{-1} PA, using 0.1 mol L^{-1} HCl (blue line), 0.1 mol L^{-1} $HClO_4$ (orange line), and BR buffer solution (pH 2.0, green line), as the supporting electrolytes. SWV conditions: (*a*): 40 mV; Δ*E*s: 70 mV; (*f*): 10 s^{-1}. **(D)** Cyclic voltammograms recorded for the first (light blue line) and second (dark blue line) scans for 0.2 mmol L^{-1} PA in the presence of 0.1 mol L^{-1} HCl. CV parameters: Scan rate: 50 mV s^{-1}; step potential: 5 mV.

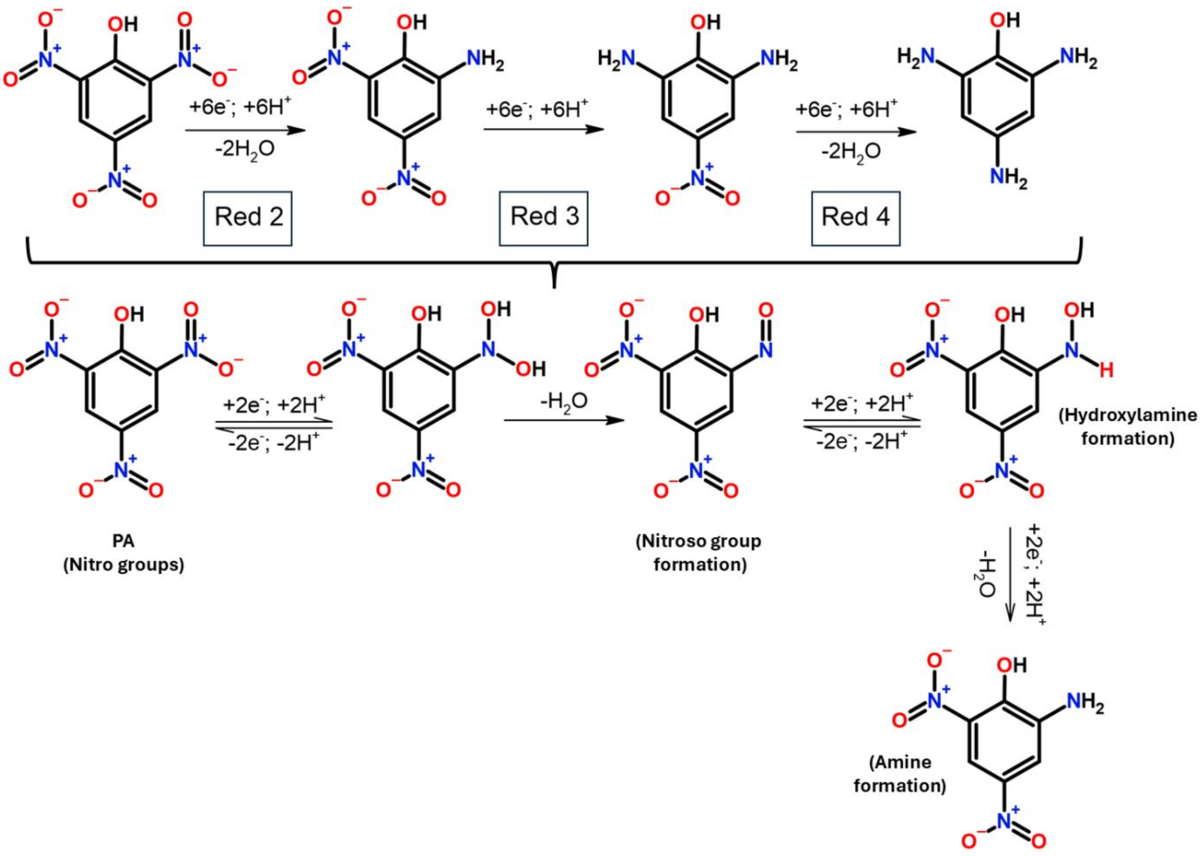

Scheme S1. Probable reduction mechanisms of PA at the C-SPE surface. The illustration shows the stepwise reduction of the three nitro groups present in the PA molecule, leading to their conversion into the corresponding amine groups.

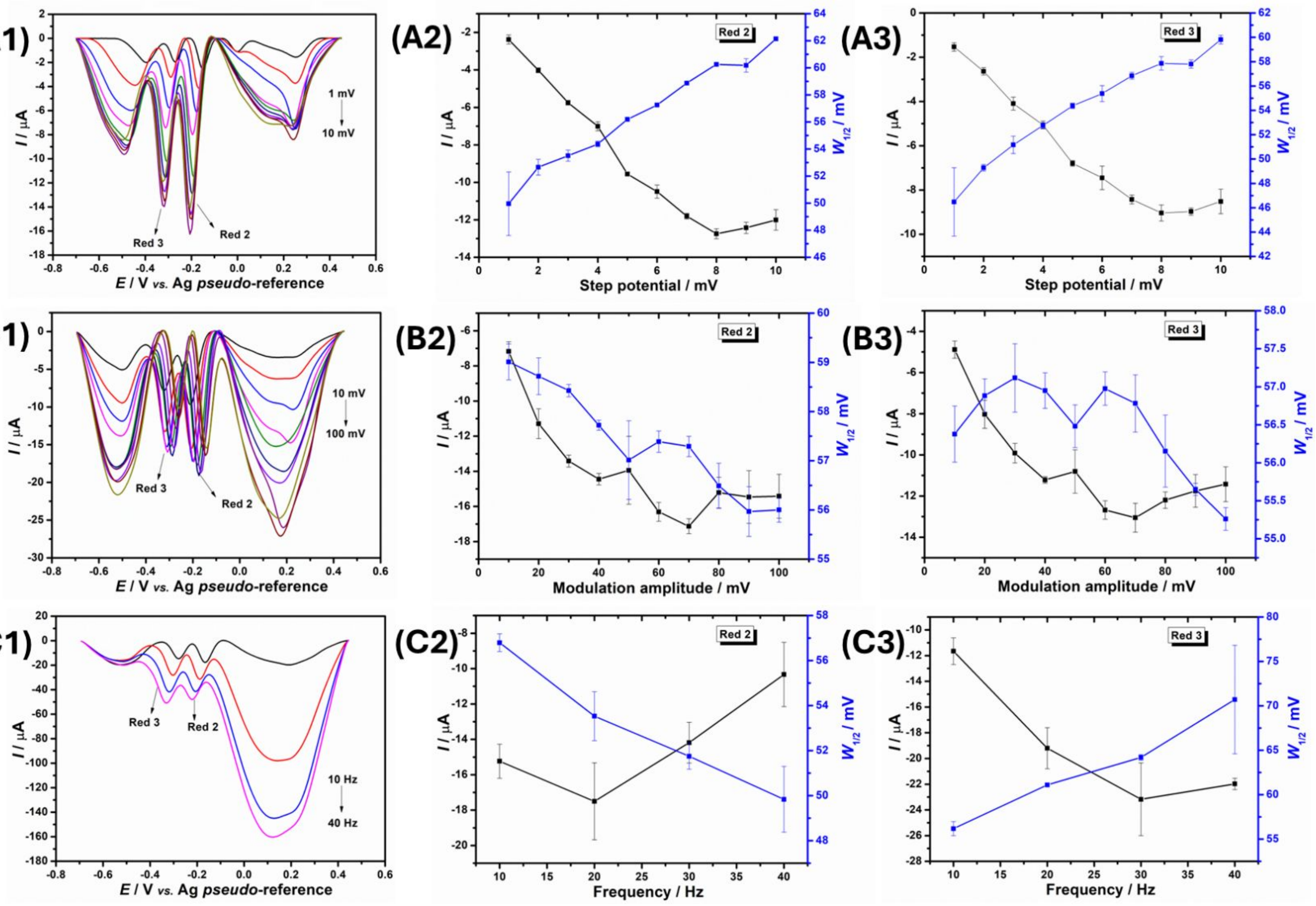

Figure S15. (A1) Baseline-corrected SWV responses and **(A2**, **A3)** are plots showing the effect of step potential on the peak current and peak half-width on Red 2 and Red 3 of PA, respectively. SWV conditions: (*f*): 10 s^{-1}; (*a*): 20 mV. **(B1)** SWV recordings, and the plots showing the impact of modulation amplitude on the peak current and peak half-width on Red 2 **(B2)** and Red 3 **(B3)** of PA. Other SWV conditions: (*f*): 10 s^{-1}; (ΔEs): 7 mV. **(C1)** SWV profiles and **(C2, C3)** are plots demonstrating the influence of frequency on the peak current and peak half-width on Red 2 and Red 3 of PA, respectively. Other SWV conditions: (*a*): 70 mV; (ΔEs): 7 mV. All voltammograms were obtained in the presence of 30 µmol L^{-1} PA using 0.1 mol L^{-1} HCl as the supporting electrolyte.

Table S4. Key analytical parameters for the determination of PA by SWV using untreated and plasma-treated C-SPEs, considering the electrochemical reduction processes Red 2 and Red 3.

Analytical parameters	Untreated C-SPE		Treated C-SPE	
	**Red 2 (PA)**	**Red 3 (PA)**	**Red 2 (PA)**	**Red 3 (PA)**
R^2	0.998	0.989	0.998	0.994
[a]Slope / µA L µmol^{-1}	-0.089 ± 0.001	-0.061 ± 0.004	-0.719 ± 0.010	-0.744 ± 0.029
[b]Intercept / µA	0.028 ± 0.009	0.247 ± 0.057	0.313 ± 0.026	0.244 ± 0.025
Linear range / µmol L^{-1}	1.0 – 50.0	10.0 – 50.0	0.5 – 50.0	0.5 – 40.0
LOD / µmol L^{-1}	0.3	2.2	0.1	0.1
LOQ / µmol L^{-1}	1.1	7.5	0.3	0.3

[a, b] Deviation obtained by linear regression.

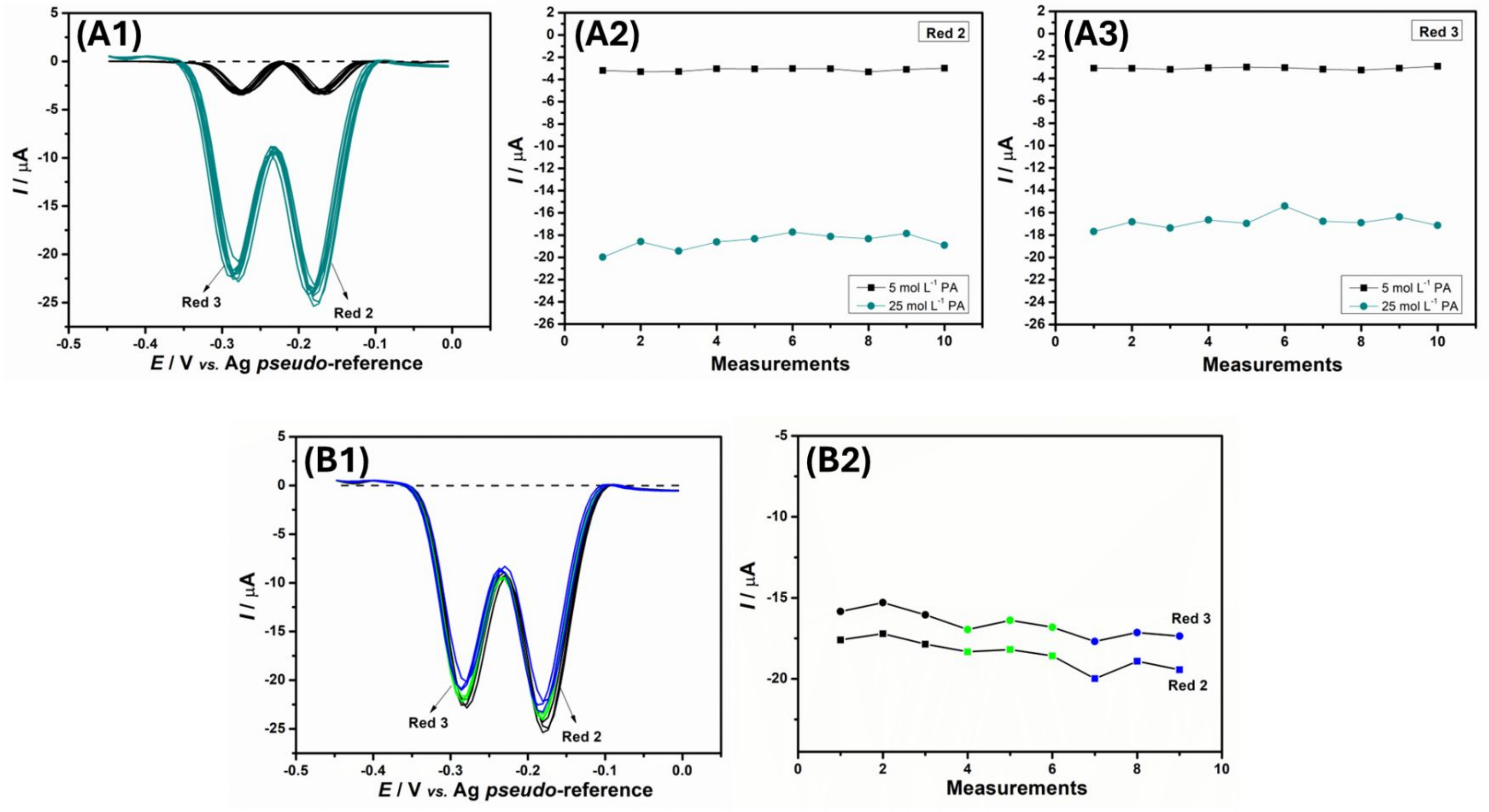

Figure S16. (A1) Baseline-corrected SWV responses for successive measurements (n = 10) of two concentration levels: 5.0 µmol L^{-1} and 25.0 µmol L^{-1} of PA (black and green lines, respectively), using a plasma-treated C-SPE. **(A2)** and **(A3)** Correspond to the peak current obtained for both reduction processes of PA (Red 2 and Red 3, respectively) by successive measurements **(B1)** Baseline-corrected SWV scans obtained in triplicate by employing three (n= 3) different plasma-treated C-SPEs (electrode 1 – black lines; electrode 2 – green lines; electrode 3 – blue lines) in the presence of 25.0 µmol L^{-1} of PA. **(B2)** Displays the peak current obtained for both reduction processes of PA by successive measurements. SWV parameters: (*f*); 10 s^{-1}; (*a*): 70 mV; Δ*E*s = 7 mV.

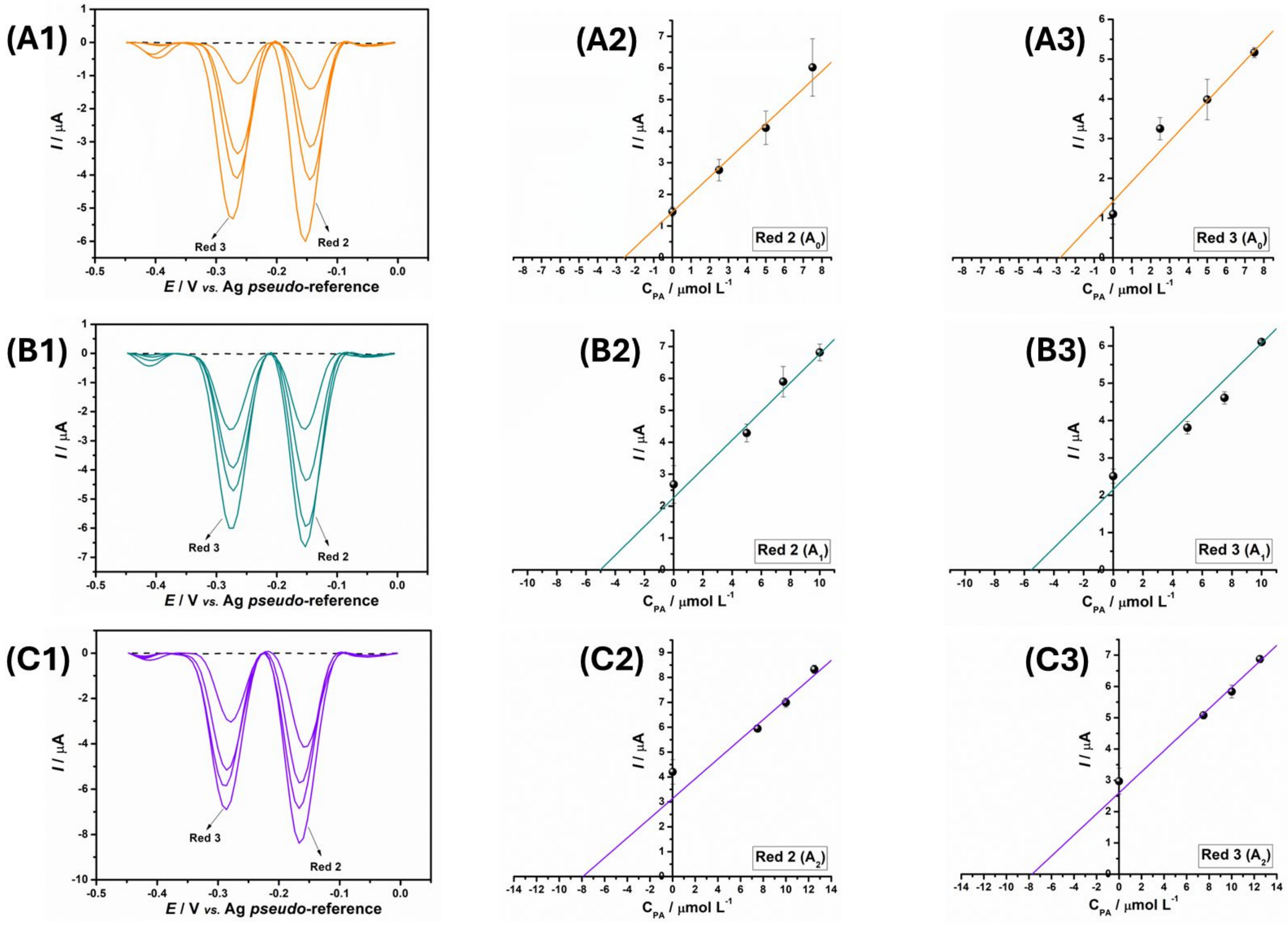

Figure S17. **(A1)**, **(B1)**, and **(C1)** show baseline-corrected SWV responses obtained from the analysis of the explosive sample (A_0, orange lines) spiked with 2.5 µmol L⁻¹ PA (A_1, green lines) and 5.0 µmol L⁻¹ PA (A_2, violet lines), respectively. The dashed lines correspond to the blank signals (0.1 mol L^{-1} HCl). **(A2)**, **(B2)**, and **(C2)** display the corresponding calibration plots constructed via the standard addition method using successive additions of PA at 2.5 µmol L⁻¹ increments, considering the Red 2 electrochemical process. In contrast, **(A3)**, **(B3)**, and **(C3)** show the calibration plots corresponding to the Red 3 electrochemical process. SWV conditions: (*a*): 70 mV; Δ*E*s: 70 mV; (*f*): 10 s^{-1}.

S3. References

(1) Siqueira, G. P.; Rocha, R. G.; Nascimento, A. B.; Richter, E. M.; Muñoz, R. A. A. Portable Atmospheric Air Plasma Jet Pen for the Surface Treatment of Three-Dimensionally (3D)-Printed Electrodes. *Anal Chem* **2024**, *96*, 15852–15858.

(2) Morales, D. M.; Risch, M. Seven Steps to Reliable Cyclic Voltammetry Measurements for the Determination of Double Layer Capacitance. *Journal of Physics: Energy* **2021**, *3* (3), 034013.

(3) Lavagnini, I.; Antiochia, R.; Magno, F. An Extended Method for the Practical Evaluation of the Standard Rate Constant from Cyclic Voltammetric Data. *Electroanalysis* **2004**, *16* (6), 505–506.

(4) Pérez-Gandarillas, L.; Aragón, D.; Manteca, C.; Gonzalez-Barriuso, M.; Soriano, L.; Casas, A.; Yedra, A. Highly Hydrophobic Organic Coatings Based on Organopolysilazanes and Silica Nanoparticles: Evaluation of Environmental Degradation. *Coatings 2023, Vol. 13, Page 537* **2023**, *13* (3), 537.

(5) Orzari, L. O.; Kalinke, C.; Silva-Neto, H. A.; Rocha, D. S.; Camargo, J. R.; Coltro, W. K. T.; Janegitz, B. C. Screen-Printing vs Additive Manufacturing Approaches: Recent Aspects and Trends Involving the Fabrication of Electrochemical Sensors. *Anal Chem* **2025**, *97* (3), 1482–1494.
